# Localized Accumulation of Tri‐n‐Propylamine Prolongs Electrochemiluminescence for Tumor Model Spheroid Analysis

**DOI:** 10.1002/anie.8508105

**Published:** 2026-06-11

**Authors:** Vanshika Gupta, Megan L. Hill, Samuel P. Nortz, Yashvi Choudhary, Ashutosh Rana, Jeffrey E. Dick

**Affiliations:** ^1^ Department of Chemistry Purdue University West Lafayette Indiana USA; ^2^ Elmore Family School of Electrical and Computer Engineering Purdue University West Lafayette Indiana USA

**Keywords:** afterglow chemiluminescence, cell spheroids, electrochemiluminescence, real‐time analysis, tris(2,2’‐bipyridyl)ruthenium(II)/tri‐n‐propylamine ([Ru(bpy)_3_]^2^^+^/TPrA), tumor analysis

## Abstract

Electrochemiluminescence (ECL) is a technique that couples electrochemical control with photon emission, enabling highly sensitive, label‐free imaging without an external light source. The microsecond lifetimes and short diffusion lengths of intermediates generated during the reaction confer excellent spatiotemporal resolution, while the absence of phototoxicity promotes biocompatibility. Previously, ECL microscopy has illuminated systems ranging from single cells to multicellular spheroids. Yet, these works relied on luminol–hydrogen peroxide chemistry, which is limited by weak signals and the sacrificial nature of luminol. Here, we introduce tris(2,2’‐bipyridyl)ruthenium(II)/tri‐n‐propylamine ([Ru(bpy)_3_]^2^^+^/TPrA) chemistry as a powerful alternative for spheroid imaging. Pre‐incubation of cellular spheroids within TPrA, followed by interrogation in [Ru(bpy)_3_]^2^^+^ produces markedly sharper spatial resolution and enables continuous imaging for over 3 h, exploiting the recyclability of the luminophore. Furthermore, this strategy reveals the first sustained afterglow chemiluminescence in a biological system, persisting for minutes after the applied potential has ended. Strikingly, we demonstrate that this methodology can elucidate differences in the ECL emission intensity in spheroids from cancerous and non‐cancerous cell lines, likely due to differences in TPrA accumulation. These advances establish [Ru(bpy)_3_]^2^^+^/TPrA‐based ECL as a transformative approach for long‐term, high‐resolution imaging of three‐dimensional cellular architectures with application towards cancerous versus non‐cancerous tumor model differentiation.

## Graphical abstract

Here, we enhance the electrochemiluminescence imaging of cellular spheroids using [Ru(bpy)_3_]^2^^+^/TPrA chemistry. Our strategy, which involves pre‐incubation with TPrA, enables high‐resolution, long‐term visualization of cellular spheroids without phototoxicity. We observe sustained afterglow chemiluminescence for minutes and demonstrate differences between cancerous and non‐cancerous spheroids. This brings forth a powerful strategy for probing three‐dimensional cellular architectures.

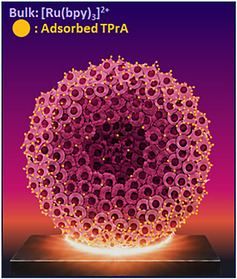

## 1 Introduction

Electrochemiluminescence (ECL) is an excited‐state electrochemical technique in which electron transfer events at the electrode surface form highly oxidizing or reducing species that subsequently react to release photons [1]. These photons can then be detected using photomultiplier tubes or highly sensitive cameras to obtain an optical intensity readout. The reaction proceeds in three stages: first, an electron is transferred between the electrode and solution‐phase species; second, these species react as they diffuse away from the electrode, forming an excited state; lastly, the excited molecule emits photons upon relaxation to the ground state [2]. Because the half‐life of these species is typically on the order of microseconds, they can diffuse only a short distance before reacting. This limitation imparts the technique with high spatiotemporal resolution and sensitivity [3]. Moreover, the absence of phototoxicity‐inducing incident light, low sample preparation time, and label‐free approach make it well‐suited for biological analysis [4, 5, 6]. Previously, ECL microscopy has been used to successfully image single cells, organelles, bacteria, and cell‐to‐matrix adhesions along with biochemical processes such as respiration, reactive oxygen species (ROS) production, and dopamine release [4, 7, 8, 9, 10, 11, 12, 13]. In addition to these systems, ECL microscopy has also been applied to complex tissue samples, such as spheroids [14]. Our group and others have recently demonstrated methods to enhance the utility of ECL in space and time, allowing one to study physicochemical properties of objects far from the electrode [15, 16] and enhancing the apparent ECL lifetime by orders of magnitude [17, 18, 19, 20].

Spheroids are three‐dimensional cellular aggregates cultivated ex vivo from traditional 2D cell cultures. They replicate many structural and functional features of living tumor tissues, including cell–cell and cell–extracellular matrix interactions, nutrient and oxygen gradients, and cellular heterogeneity [21, 22]. Due to the compact cellular arrangement, nutrients and oxygen diffuse inward due to consumption, while metabolic waste products diffuse outward, creating opposing gradients. In spheroids larger than ∼400 µm in diameter, these gradients establish three distinct microenvironments: an outer proliferation zone containing actively dividing cells, a middle quiescent zone of nonproliferating cells, and a central necrotic core marked by hypoxia, waste accumulation, acidic pH, and elevated levels of ROS [23]. Despite being grown ex vivo, this organization allows for spheroids to mimic the extracellular complexity, cellular heterogeneity, and nutrient gradients present in tissue microenvironments. Thus, spheroids are gaining attention as possible replacements for animal models in fields such as neuroscience, pharmacology, and toxicology [24]. Furthermore, there is growing evidence that in vitro 3D cell models, particularly organoids [25, 26], serve as a more representative model for real human tissue than in vivo animal models. Spheroids are also promising for cancer studies, as spheroids grown from cancerous cells can effectively mimic malignant tumors [27]. However, despite their promising outlook, spheroids have been characterized by relatively few methods, such as fluorescence microscopy and mass spectrometry [22, 28]. Moreover, most of these methods require long sample preparation times and often call for the homogenization of the entire spheroid sample, which not only precludes any real‐time studies on these models but also results in a loss of key spatial and temporal biochemical information that could potentially inform pharmaceutical and biomedical investigations. Therefore, complementary techniques are needed that can visualize these models with good spatiotemporal resolution. Recently, our group and the Shiku group have advanced the adaptation of ECL microscopy for studying these complex three‐dimensional tumor models. Specifically, we visualized endogenously produced hydrogen peroxide in cancerous and non‐cancerous spheroids, while the Shiku group evaluated the respiratory activity of mesenchymal stem cell spheroids [14, 29]. These proof‐of‐concept studies employed the well‐known luminol–hydrogen peroxide system to establish ECL as a valuable tool for spheroid analysis; however, they were limited by the low hydrogen peroxide concentrations released from the spheroid and the sacrificial nature of luminol, which reduced the spatial resolution [30].

In this work, we establish the mechanistically well‐known reactant–coreactant pair tris(2,2’‐bipyridyl)ruthenium(II) ([Ru(bpy)_3_]^2+^)–tri‐n‐propylamine (TPrA) for imaging actively respirating spheroids. By pre‐incubating spheroids in a TPrA solution prior to interrogation in a solution of [Ru(bpy)_3_]^2+^, we markedly enhance spatial resolution while leveraging the recyclability of [Ru(bpy)_3_]^2+^ to enable imaging for over 3 h as TPrA is gradually released from the spheroid. Equally significant, we report a sustained luminescence that persists up to 180 s after cessation of the applied potential demonstrating the first observation of afterglow chemiluminescence in a biological system, to our knowledge. Afterglow chemiluminescence is broadly defined as the emission of light that persists long after a potential bias is no longer applied to a system. Our system is an example of what can be defined as “ECL‐initiated, coreactant‐release‐based afterglow chemiluminescence,” as the signal is initiated by the bulk oxidation of [Ru(bpy)_3_]^2+^ to [Ru(bpy)_3_]^3+^ during the ECL that occurs before the afterglow chemiluminescence, and driven by the release of TPrA, the coreactant, from the spheroid. Moreover, to our knowledge, this is the first reported afterglow chemiluminescence system enabled by the release of a coreactant. We also demonstrate that the [Ru(bpy)_3_]^2+^–TPrA system can reveal differences between spheroids derived from cancerous versus non‐cancerous cell lines through variations in the ECL intensities, likely due to differing extents of TPrA accumulation in the spheroids. Additionally, we can visualize differences in the spheroid–electrode interface across spheroids from various human cell lines based on the distinct ECL emission layer diffusion profile for each cell line. These findings establish this [Ru(bpy)_3_]^2^^+^/TPrA‐based ECL methodology as a cutting‐edge approach for long‐term, high‐resolution interrogation of three‐dimensional cellular architectures. Moreover, like other relatively non‐destructive techniques, such as the MasSpec Pen [31], our methodology shows promise in distinguishing healthy from cancerous tissue.

## 2 Results and Discussion

ECL microscopy using the luminophore [Ru(bpy)_3_]^2+^ and the sacrificial coreactant TPrA has previously allowed for the successful imaging of biological samples predominantly using an ECL technique known as shadow ECL [4] in which the biological entity remains unlabeled with [Ru(bpy)_3_]^2+^and TPrA suspended in bulk solution. Upon application of potential, which initiates and sustains the ECL emission, the background is illuminated while the sample itself remains shadowed due to its insulating properties. This technique is advantageous as it allows for the analysis of cellular morphology, and requires little sample preparation aside from the upkeep of the biological entity. However, the contrast mechanism precludes the technique from providing direct information regarding the reactions happening at the cell membrane | electrode interface. As a solution, we propose a unique ECL protocol in this study that allows for the illumination of the spheroid while the background of the system remains largely dark to yield a “positive image” synonymous with positive ECL without the need for arduous functionalization and washing steps. This is advantageous because by limiting photon emission to the sample, the spatial resolution of the surface of the sample is greatly enhanced, and the contrast between the background and the sample itself is maximized. Often, this type of ECL imaging is achieved through the immobilization of the [Ru(bpy)_3_]^2+^ luminophore to the biological sample via a covalently bound streptavidin tag while the co‐reactant TPrA is in the bulk solution [13]. In this work, we advance this ECL technique by developing a method where we can achieve the illumination of the biological sample, a spheroid, without the immobilization of the luminophore. We use this ECL method to visualize the spheroid‐electrode interface seeking to elucidate biochemical information.

**FIGURE 1:**
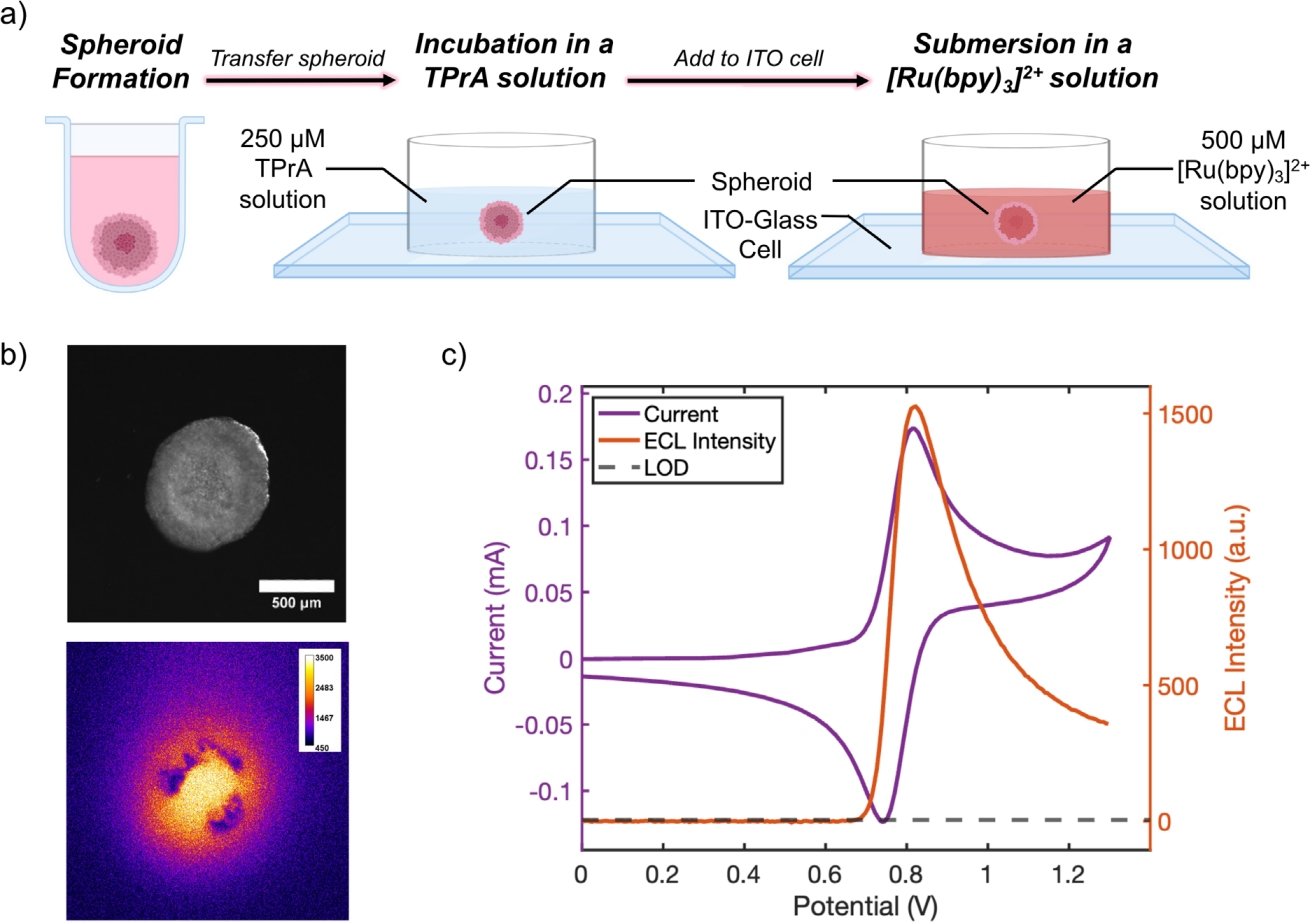
(a) Schematic showing the spheroid incubation and interrogation process with ECL microscopy. In the first step, spheroids are grown; then, in the second step, they are transferred to a solution of 250 µM TPrA and 25 mM glucose within 1× DPBS. The spheroid is incubated in this solution for 15 min, then transferred into an ITO‐glass cell containing a solution of 500 µM [Ru(bpy)_3_]^2+^ and 25 mM glucose within 1× DPBS, where the spheroid is then interrogated using ECL microscopy. (b) Brightfield micrograph (top) and ECL micrograph (bottom) of an MCF‐7 spheroid imaged using the methodology described in (a). (c) Correlated cyclic voltammetry with ECL intensity obtained as a response to potential application. The limit of detection (LOD) was calculated by taking the standard deviation of the first 15 frames of the background signal (before potential was applied) and multiplying it by a factor of 3.

In a typical experiment, we first grew MCF‐7 breast cancer spheroids from a two‐dimensional cell culture. We chose these cells as initial test subjects because they are a well‐established cell line and have previously been shown to yield reproducible spheroid morphologies. To generate these spheroids, we grew MCF‐7 cells to an optimal cell density of 1 × 10^6^ cells per plate, from which we plated 2 × 10^4^ cells in each well of an ultra‐low attachment 96‐well plate to form spheroids. We allowed these spheroids to mature for 7 days until we could visualize three distinct layers, expressed as different shades of pink in Figure 1a, and we could observe an optimal spheroid size of 500 ± 55 µm using an optical microscope. Additionally, we assembled an electrochemical cell by attaching a glass cylinder to an optically transparent indium tin oxide (ITO) working electrode using commercially available epoxy to hold the ECL solution, a silver (Ag) wire quasi‐reference electrode, and a glassy carbon rod counter electrode. We placed this three‐electrode cell atop an inverted microscope functionalized with an EMCCD camera to record ECL images as well as a CMOS camera to acquire brightfield images. We used a 4× long working distance objective with a 1.0× modifier internal to the microscope, and we controlled the electrochemistry using a CH Instruments potentiostat. We conducted data acquisition and microscope control using a computer (Figure S1).

To benchmark the [Ru(bpy)_3_]^2+^–TPrA system for spheroids, we sought to replicate the commonly used shadow ECL setup where the analyte solution containing both the luminophore (500 µM [Ru(bpy)_3_]^2+^) and the coreactant (250 µM TPrA) was added to an ITO glass cell, as shown in Figure S2A, along with the quasi‐reference and counter electrodes. Next, we obtained a brightfield image to visualize the spheroid using a CMOS camera (Figure S2B), followed by the application of a constant potential at 0.8 V versus a silver wire quasi‐reference electrode for 180 s to carry out the ECL microscopy (Figure S2C,D). We show the current versus time curve for this experiment in Figure S2E. For all the experiments we describe in this study, we record the ECL intensity along with the electrochemical current trace using an EMCCD camera and a potentiostat, respectively, during the potential application. As expected, the combined solution of [Ru(bpy)_3_]^2+^–TPrA resulted in an image with a bright background, which is characteristic of shadow ECL experiments. In typical shadow ECL experiments, freely diffusing luminophore species are excited at the electrode surface in all areas except those covered by cells [32]. When these excited species react with the TPrA in bulk solution, light is emitted at the electrode surface, allowing for the visualization of key morphological details of the insulating cells as well as a clear distinction of their boundaries.

In our experiments, we were able to see regions of the spheroid that showed illumination characteristic of shadow ECL. Specifically, we observed small dark patches that were typically ∼10 µm in size at the spheroid surface. We infer that these patches are attachment points, where the cells at the bottom of the spheroid adhere to the ITO electrode. At the adhesion points, the cells likely act as insulators, blocking the electrode surface, resulting in the hindrance of the luminophore's access to the electrode surface beneath, akin to that observed in shadow ECL. At the same time, we see large areas of illumination at the center of the spheroid with intensities close to those of the bulk solution. This high intensity at the spheroid surface we observed is similar to that seen in positive ECL systems, where the luminophore is typically covalently bound to the biological species, while the TPrA is allowed to diffuse freely. In such a case, because access to the luminophore and coreactant stays constant and unhindered, unlike in shadow ECL, surface features of the cells can be observed. In our initial experiments, both the luminophore and coreactant were present in bulk solution. We anticipated that in these experiments, the whole spheroid should act like an insulator, blocking the signal, while the ECL intensity of the background or bulk solution would be high due to the freely diffusing reactants. However, we did not observe this. One explanation for this visual phenomenon could be the release of biological coreactants from the spheroid itself. However, if this were the case, the entirety of the spheroid would show ECL intensities higher than the bulk, excluding the adhesion points. Another key point to note is that Mockaitis et al. showed the diffusion of biological coreactants to extend ∼170 µm away from the spheroid, while in the current system, ECL is confined to the center of the spheroid, with no observable light intensity gradient around the spheroid [14]. Instead, this behavior could indicate a varying surface topography of the spheroid moving away from the electrode surface, which might display concave areas where one of the reactants could be trapped during the reaction, generating a profile wherein the spheroid appears to be emitting light. This variation in surface is typical of tissue cultures grown ex vivo and it arises from the lack of attachment surfaces during the growth of the spheroid.

To investigate, we separately introduced the two components of the system to the spheroid by exposing the spheroid to each reactant sequentially. First, we incubated the spheroid in a solution of 500 µM [Ru(bpy)_3_]^2+^ for 15 min. Next, we removed the spheroid from the solution and placed it in a solution of 250 µM TPrA, where we conducted the experiment. The brightfield images and corresponding ECL micrographs are shown in Figure S3. Similarly, we investigated the reverse system, where we incubated the spheroid in 250 µM TPrA for 15 min, removed it from the solution, and then immersed it in a solution of 500 µM [Ru(bpy)_3_]^2+^ in which we applied the voltage. Figure 1a depicts this process. The brightfield micrograph and the ECL micrograph are seen in Figure 1b (top and bottom images, respectively). Overall, the first scenario resulted in very low ECL intensity, indicating that a small fraction of [Ru(bpy)_3_]^2+^ may accumulate in the spheroid. This accumulated luminophore concentration could leak onto the surface of the electrode where we observed ECL due to the presence of the components necessary for ECL to take place: luminophore, coreactant, and electrode. Conversely, this observed ECL intensity could have also been caused by the transfer of [Ru(bpy)_3_]^2+^ from the incubation solution into the ECL interrogation solution. To test if ECL arises due to the accumulation of [Ru(bpy)_3_]^2+^ within the spheroid or from a different mechanism, we studied spheroids from two other cell lines, one of the cell lines being non‐cancerous, HPNE, and the other being cancerous, Panc1. As with the spheroid from the MCF‐7 cell line, we incubated each of these spheroids in a solution of 500 µM [Ru(bpy)_3_]^2+^ for 15 min. Then, we transferred the spheroids to a 1x DPBS solution for brightfield and fluorescence microscopy. In fluorescence microscopy, we used an excitation wavelength of 550 nm to excite any retained [Ru(bpy)_3_]^2+^, and a bandpass filter (604–679 nm) to limit the accepted wavelengths to the EMCCD detector to the expected approximately 620 nm emission wavelength of [Ru(bpy)_3_]^2+^ [18]. Following this, we performed ECL microscopy in a solution of 250 µM TPrA, holding a potential of 1.1 V versus Ag wire for 180 s. For a discussion on why we held 1.1 V versus Ag wire for these experiments, see Supplemental Note 1 and Figure S4. Figure S5 includes the results of this experiment for the spheroids from the HPNE cell line, while Figure S6 summarizes the results of this experiment for the spheroids from the Panc1 cell line. As can be seen in Figures S5 and S6, when we incubate the spheroids in [Ru(bpy)_3_]^2+^, there is a positive fluorescence signal within the spheroids, indicating that some amount of [Ru(bpy)_3_]^2+^ is accumulated. However, we did not observe an ECL signal from either spheroid, suggesting that the accumulated [Ru(bpy)_3_]^2+^ does not contact the electrode, which prevents ECL from taking place. We hypothesize that this could be caused by a very low concentration of [Ru(bpy)_3_]^2+^ accumulating within these spheroids. This [Ru(bpy)_3_]^2+^ likely leaches out of the spheroid but its resulting illumination is not bright enough to be monitored by our system.

However, in the reverse system, where we first incubate the spheroid in TPrA, we see behavior akin to the non‐incubated system except for the elimination of the background signal. The background we observed in this situation is nearly devoid of intensity, while most of the spheroid surface shines brightly, hence enhancing the contrast between the background and the sample of interest. Additionally, we can distinguish the adherence points of the spheroid more clearly compared to those in Figure S3C, with sizes representative of either average MCF‐7 cells (19.9–33.9 µm on average) [33] or adjacent groups of cells crowded together. Thus, our sequential reactant exposure technique not only allows for the visualization of the spheroid–electrode interface in general but can also resolve clusters of cells at the spheroid–electrode interface. Notably, we hypothesize that this high ECL intensity at the spheroid–electrode interface indicates that the spheroid can accumulate enough TPrA to mimic bulk concentrations of the coreactant in a relatively short incubation timeframe and can do so without the need for covalent tagging.

To further interrogate this new ECL methodology, we conducted control experiments to validate that the ECL emission observed is solely due to the ECL reaction between the [Ru(bpy)_3_]^2+^ and TPrA. ECL was performed in a 500 µM [Ru(bpy)_3_]^2+^ only solution (Figure S7) and, separately, a 250 µM TPrA only solution (Figure S8). It is important to note that we observed a faint ECL intensity in the [Ru(bpy)_3_]^2+^ only solution, likely due to the presence of biological coreactants, as shown by Mockaitis et al. [14], while the TPrA solution alone yielded no measurable signal. Moreover, to probe the system electrochemically and further verify that the luminophore is in fact [Ru(bpy)_3_]^2+^, we interrogated spheroids using cyclic voltammetry (CV). We conducted a current sweep from 0 to 1.3 V at a scan rate of 0.05 V/s and recorded the resulting current as a function of the applied potential. At the same time, we used the EMCCD camera to image the resulting ECL intensity. Figure 1b shows the brightfield image of the spheroid (top) and the corresponding ECL micrograph (bottom). Comparing the ECL and the CV in Figure 1c, we can see that while the onset of ECL occurs around ∼0.7 V, interestingly, we observe the highest intensity at 0.8 V. The intensity then rapidly diminishes as the voltage is swept further in the positive direction. This indicates that this voltage is ideal for the analysis of spheroids using our sequential exposure ECL system, and it also supports that the luminophore is [Ru(bpy)_3_]^2+^, as the CV is characteristic of [Ru(bpy)_3_]^2+^ oxidation. Specifically, the current trace observed in the system mimics that of the oxidation of [Ru(bpy)_3_]^2+^ alone, as evidenced by the symmetric shape of the voltammogram that demonstrates good reversibility of the reaction as compared to the [Ru(bpy)_3_]^2+^/TPrA bulk system, where the CV is not symmetrical because of the non‐reversible oxidation of TPrA [34]. This is likely because only a very small portion of the electrode (∼500 µm) at the electrode | spheroid | solution interface interacts with the TPrA accumulated in the spheroid, with the bulk of the reaction occurring on the electrode surface being the reversible oxidation of [Ru(bpy)_3_]^2+^ species.

To optimize this methodology, we carried out investigations to determine the lowest achievable TPrA concentration and exposure time for the spheroids without significantly dampening the ECL intensity. TPrA, as a reagent, is known to have low biological compatibility and is toxic to cells [35, 36]. While literature does not provide an exact concentration that might significantly change spheroid function and viability, it was important to consider any cytotoxic effects the cells might be undergoing due to TPrA exposure. Thus, we performed an all/dead fluorescence study with Hoechst as the first stain (blue emission) that tags the nuclear membrane of all cells and propidium iodide as the dead stain (red emission) that penetrates through the cell membranes of only the dead cells (Figure S9). We saw little to no cell death for the untreated, 125 µM TPrA, and 250 µM TPrA spheroids, while we observed a substantial impact on cellular viability at 500 µM TPrA and 750 µM TPrA. Thus, 125 µM is optimal for the experiments from a viability standpoint. However, when probed using the sequential exposure ECL system, this concentration resulted in poorly defined ECL images (Figure S10A). In contrast, 250 µM (S10B), 500 µM (S10C), and 750 µM (S10D) yielded high contrast ECL images, with the largest concentration allowing for the highest emission intensity. As a compromise between the ECL signal intensity and resolution, as well as the cell viability, we identified 250 µM TrPA to be the optimal concentration for the incubation step. Additionally, to explore the influence of the TPrA incubation on the ECL imaging, we varied the incubation time between 5 min and 15 min, with the latter showing the highest quality ECL imaging (Figure S11). It is important to highlight that the independence of this methodology from the TPrA concentration is advantageous because it allows for the minimization of coreactant‐induced toxicity to the cells.

**FIGURE 2:**
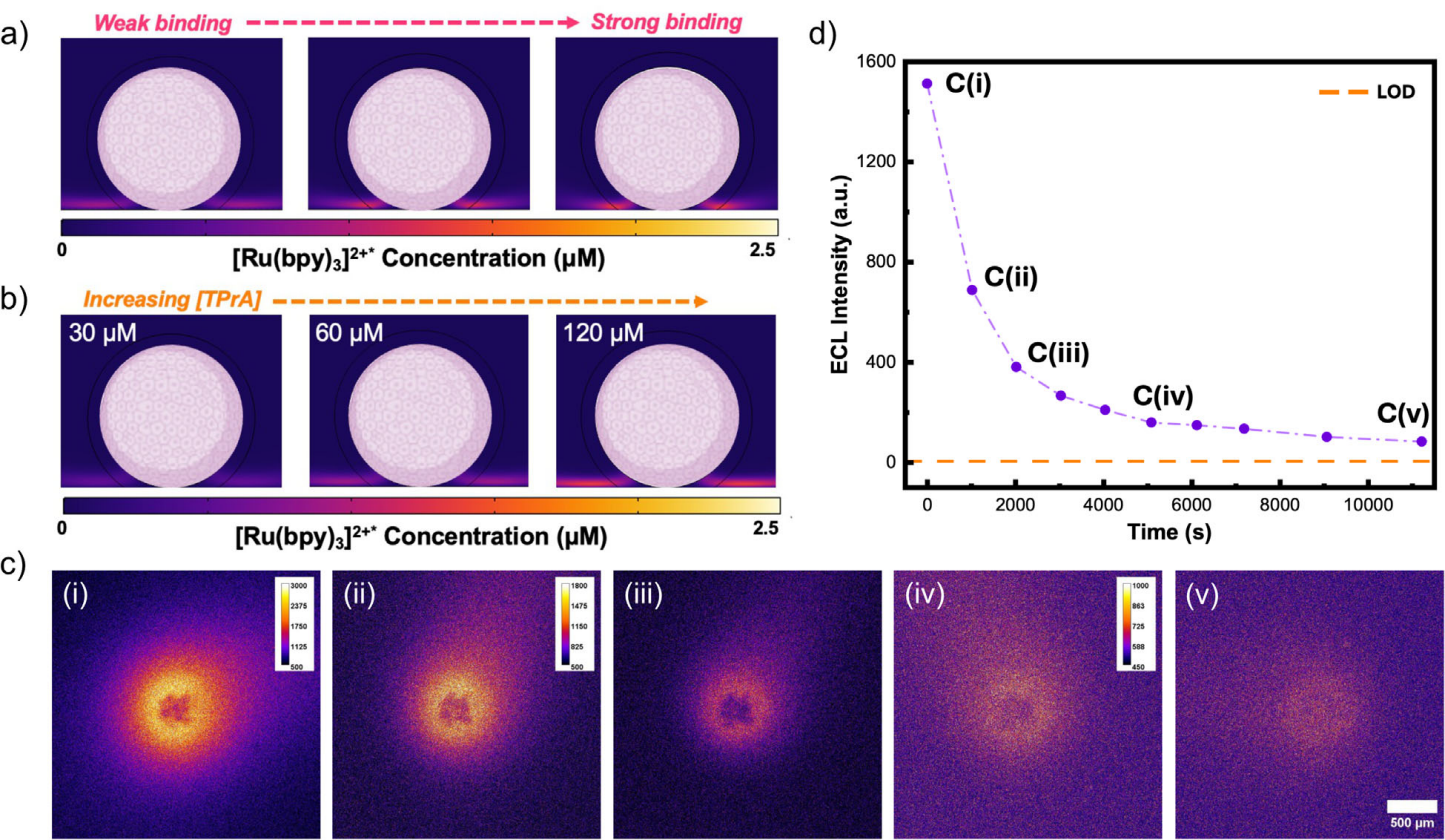
Exploring TPrA accumulation and release from a spheroid observed over time with finite‐element modeling and experimentation. (a) Finite‐element simulation of differing TPrA release kinetics from a spheroid at 180 s into the application of potential. The TPrA release kinetics were altered by assuming different diffusion coefficient values for TPrA diffusion out of and away from the spheroid. The left image has *D*_TPrA_ = 5 × 10^−11^ m^2^/s, the central image has *D*_TPrA_/100 (or 5 × 10^−13^ m^2^/s), and the right image has *D*_TPrA_/10 000 (or 5 × 10^−15^ m^2^/s) [10]. (b) Finite‐element simulation of varied TPrA accumulation within the spheroid. The left image presumes a concentration of 30 µM TPrA in the region immediately surrounding the spheroid, while the center image presumes 60 µM TPrA, and the right image presumes 120 µM TPrA. (c) ECL micrographs obtained from the CVs that were collected over different time points of the spheroid being in the 500 µM [Ru(bpy)_3_]^2+^ solution. All scale bars = 500 µm. (d) Decrease in ECL intensity as the spheroid remains in the 500 µM [Ru(bpy)_3_]^2+^ solution over time, with CVs recorded periodically. Each of the purple dot represents the maximum ECL intensity of a CV. The orange dashed line indicates the LOD.

Historically, ECL microscopy is carried out with a higher concentration of coreactant as compared to the concentration of the luminophore to ensure that the reaction is not limited by coreactant concentration [1]. However, for our system, the incubation solution concentration of TPrA is only 250 µM, less than its [Ru(bpy)_3_]^2+^ luminophore concentration of 500 µM. We hypothesize that the coreactant is readily absorbed by the spheroid, limiting the ECL signal largely to the spheroid–electrode interface, and potentially resulting in ECL intensities that mimic a higher concentration of TPrA than is present. The theorized rapid absorption of TPrA is likely driven by the coreactant's hydrophobicity and limited solubility in aqueous solutions. TPrA is an organic reagent with only ∼5 mM solubility in water [37]. Thus, in the presence of the spheroid, which is made up of cells with lipid cell membranes, the TPrA greatly favors localization at these lipid bilayers through hydrophobic interactions as opposed to being in the aqueous bulk solution. It is important to note that these aforementioned hydrophobic interactions are generally transient in nature. Therefore, the TPrA might dissociate and leak into the solution over time to generate a concentration gradient. To investigate this TPrA retention and release from the spheroid, we performed finite‐element simulations to evaluate how diffusion and local concentration influence the observed ECL response (Figure 2). In these simulations, we modulated the rate of TPrA release by using a thin‐layer boundary condition outside the spheroid with a variable diffusion coefficient to mimic differing release kinetics. Additionally, we modeled the extent of accumulation by adjusting the local TPrA concentration in the interfacial region. For a description of how we composed the finite‐element model and how we carried out the simulations, see Supplemental Note 2, Table S1, and Figure S12.

The simulations also provide insight into the role of the spheroid as a physical barrier to electron transfer. In regions where the spheroid is in direct contact with the electrode, no excited state [Ru(bpy)_3_]^2^^+^* is generated, indicating the absence of ECL emission (Figure 2a). This behavior is consistent with the spheroid acting as a blocking object, in which the insulating cellular structure prevents redox‐active species from accessing the electrode surface and undergoing electron‐transfer reactions. This prediction is in direct agreement with experimental ECL micrographs, where we observe dark regions at the spheroid surface that we attribute to points of contact between the spheroid and the electrode. In these regions, [Ru(bpy)_3_]^2^^+^ cannot be oxidized to [Ru(bpy)_3_]^3^^+^, and species generated elsewhere on the electrode surface are unlikely to diffuse into these confined regions, thereby preventing ECL emission.

Beyond these spatial effects, the simulations reveal that both TPrA release kinetics and accumulated TPrA concentration influence the ECL response in distinct ways. Varying the diffusion coefficient of TPrA over multiple orders of magnitude primarily affects the spatial confinement of the emission layer, with slower diffusion leading to more localized ECL near the spheroid–electrode interface. Notably, reducing the diffusion coefficient from 5 × 10^−13^ m^2^ s^−^^1^ to 5 × 10^−15^ m^2^ s^−^^1^ results in minimal changes to the overall ECL intensity at 180 s (Figure 2a and Figure S13), indicating that release kinetics alone do not govern signal magnitude.

In contrast, the concentration of accumulated TPrA exerts a strong influence on ECL intensity. Increasing the local TPrA concentration from 30 µM to 60 µM and then to 120 µM leads to pronounced increases in simulated emission intensity (Figure 2b and Figure S14), consistent with a greater availability of coreactant to sustain the catalytic pathway of ECL. These results suggest that while diffusion controls the spatial distribution of emission, the extent of TPrA accumulation within the spheroid is the dominant factor governing signal intensity.

To experimentally probe the temporal evolution of TPrA release and its impact on ECL intensity, a single spheroid was interrogated periodically over 3.5 h using cyclic voltammetry coupled with ECL imaging. Measurements were performed every 15–30 min. The corresponding ECL micrographs at 0.8 V from each interrogation are shown in Figure 2c, with the extracted intensity plotted as a function of time in Figure 2d. Representative cyclic voltammograms are provided in Figure S15. The ECL intensity at the spheroid interface decreases sharply during the first hour of interrogation before reaching a quasi‐steady state. Notably, even after ∼3 h (≈11 200 s), the spheroid remains clearly distinguishable from the surrounding solution, as shown in Figure 2c(v), indicating persistent localization of emission. Concurrently, the ECL intensity in the bulk solution increases over time, consistent with the gradual release of TPrA from the spheroid into the surrounding electrolyte.

These experimental observations are in strong agreement with the finite‐element simulations, supporting a mechanism in which the spheroid acts as a reservoir that gradually releases TPrA over time. The initial rapid decrease in localized intensity reflects depletion of readily accessible TPrA near the interface, while the subsequent steady‐state regime suggests a balance between continued release and diffusion into the bulk solution. Together, these results further reinforce the role of TPrA accumulation and release in governing both the temporal and spatial characteristics of the ECL response and provide a mechanistic basis for the experimentally observed differences in ECL intensity across spheroids derived from different cell lines.

We explored this TPrA release mechanism further by extracting the spheroid post‐experiment and interrogating the solution again using the same CV parameters (Figure S16). While not as bright in the absence of the spheroid, we observed an appreciable ECL intensity above the baseline in the solution. To ensure this emission intensity comes solely from the TPrA released from the spheroid instead of from [Ru(bpy)_3_]^2+^, we performed ECL in a solution of 500 µM [Ru(bpy)_3_]^2+^, using the same experimental setup as shown in Figure S1, except without a spheroid. As can be seen in Figure S17, we did not observe any ECL emission above the limit of detection for both an amperometric *i*–*t* curve held at 1.1 V versus Ag wire for 180 s and a CV swept from 0 to 1.3 V versus Ag wire, indicating the ECL most likely stems from TPrA released into solution. We hypothesize that the ECL signal observed in Figure S16 could arise from both the release of TPrA from the spheroid into the solution, as well as from TPrA that we may have transferred into the ITO cell when we removed the spheroid from the TPrA incubation solution and placed it into the [Ru(bpy)_3_]^2+^ ECL solution. However, we expect nearly all the concentration of the coreactant that we may have transferred into the ECL solution from the incubation solution to have been used up in the initial part of the reaction when the spheroid was still in solution. Hence, the observed ECL signal is most likely to be from TPrA release by the spheroid.

To understand the depth to which TPrA is absorbed by the spheroid, we conducted a serial trypsinization experiment such that after the correlated ECL‐voltammetry study, we exposed the spheroid to the common enzyme trypsin. Trypsin is known for its ability to cleave peptide linkages between cells [22, 38, 39]. This treatment disassociated the first few cellular layers, which we then collected and interrogated separately in a 500 µM [Ru(bpy)_3_]^2+^ only solution. We present the results of these investigations in Figure S18. While both fractions yielded intensities that were lower than those of the original investigation, the ECL we observed in the cell layer aliquot was still greater than that of the treated spheroids. Moreover, the single cells showed a true shadow ECL profile such that the surface of the cells remained dark and the bulk solution yielded ECL. These observations support our hypotheses that TPrA's interaction with the cell membrane is transient and that TPrA accumulates in the spheroid. Moreover, it supports the idea that TPrA accumulates primarily within the first few layers of the spheroid, making it most accessible to the [Ru(bpy)_3_]^2+^ in the bulk solution.

In addition to the TPrA concentration, we also optimized the [Ru(bpy)_3_]^2+^ concentration. To do so, we interrogated spheroids in 125, 250, 500, and 750 µM [Ru(bpy)_3_]^2+^ while keeping a constant TPrA incubation concentration of 250 µM (Figures S19 and S20). All concentrations show the previously described symmetrical voltammogram with increasing peak currents as a function of increasing concentration (Figure S19). Furthermore, we observed somewhat similar maximum ECL intensities for 500 and 750 µM [Ru(bpy)_3_]^2+^, while we saw a sharp rise in maximum ECL intensity when we increased the concentration from 250 to 500 µM. To further probe the difference between the ECL intensity for the 500 and 750 µM [Ru(bpy)_3_]^2+^ systems, we performed amperometry experiments by holding a voltage of 0.8 V. The resulting current versus time graphs showed a higher current magnitude for 750 µM [Ru(bpy)_3_]^2+^ as expected (Figure S20). When comparing the ECL intensities using this technique, the 750 µM [Ru(bpy)_3_]^2+^ solution clearly showed the highest ECL intensity. Interestingly, its intensity trace was quite different from the other concentrations, such that while its signal stabilized initially at the 50 s timepoint like the others, the glow intensified upon extended potential application. This could be due to several factors, including liberation of TPrA from cells further inside the spheroid, lysing of the cells due to continued voltage application, or reactive oxygen species induced stress due to continued exposure to exogenous mediators as shown by Nortz et al. [40]. Thus, to minimize perturbation to cellular homeostasis, we chose 500 µM [Ru(bpy)_3_]^2+^ as the optimal concentration.

**FIGURE 3:**
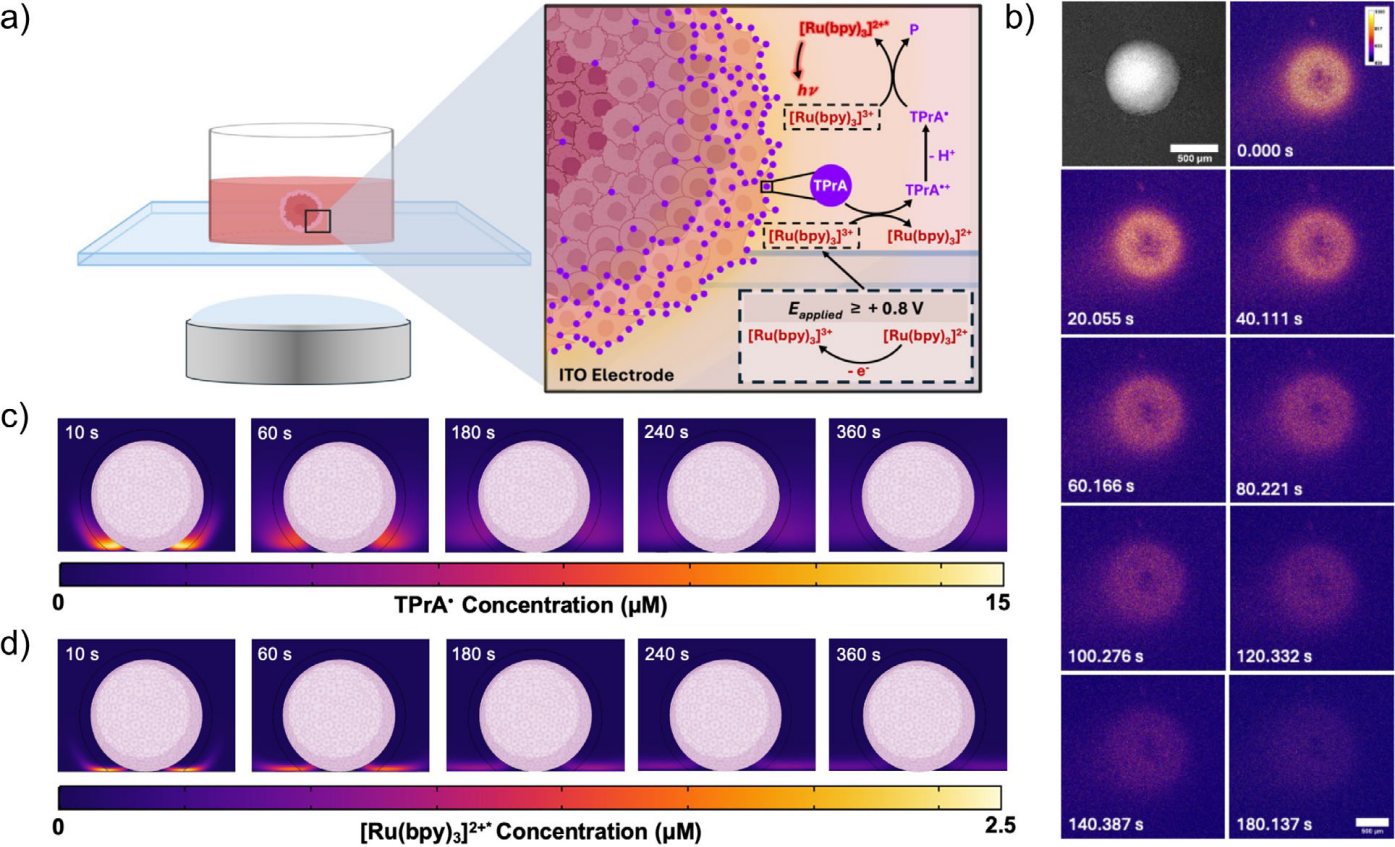
(a) Schematic showing the experimental setup with the ITO cell above the microscope objective and the slow release of TPrA from the MCF‐7 spheroid. The schematic also includes the proposed ECL and afterglow chemiluminescence mechanism, known as the catalytic route. (b) Brightfield image of an MCF‐7 spheroid followed by ECL micrographs obtained after the cessation of an applied potential. A potential of 0.8 V versus an Ag wire was held for 180 s prior to the afterglow chemiluminescence being observed. The time of *t* = 0.000 s denotes the first frame of afterglow chemiluminescence after the cessation of the applied potential. As shown with the micrographs, afterglow chemiluminescence can be seen on the spheroid for approximately 180 s after the applied potential stops. All scale bars = 500 µm. (c) Finite‐element simulation demonstrating the concentration profile for the TPrA^•^ during both the application of potential for 180 s (images at 10, 60, and 180 s) and in the afterglow chemiluminescence regime (images at 240 and 360 s). (d) Finite‐element simulation demonstrating the concentration profile for the [Ru(bpy)_3_]^2+*^, excite state [Ru(bpy)_3_]^2+^, during both the application of potential for 180 s (images at 10, 60, and 180 s) and in the afterglow chemiluminescence regime (images at 240 and 360 s). Both simulations assumed a TPrA concentration of 60 µM in the areas immediately surrounding the spheroid and a bulk [Ru(bpy)_3_]^2+^ concentration of 500 µM.

Another key observation from this current versus time study was that of an extended glow after the end of the application of a potential. Figure 3 shows this phenomenon through snapshots of ECL over time (Figure 3b), with the current trace for this experiment shown in Figure S21, and presents a model for this afterglow chemiluminescence emission (Figure 3a). It is important to highlight that this work marks the first‐ever reported observation of the phenomenon called “afterglow” chemiluminescence with a biological sample, to our knowledge. Afterglow chemiluminescence has previously been demonstrated by our group as well as the groups of Francisco Del Campo and Shu‐Feng Zhou in [Ru(bpy)_3_]^2+^ only, [Ru(bpy)_3_]^2+^‐persulfate anion, luminol‐H_2_O_2_, and CN_x_ nanosheet system, respectively [15, 17, 18, 41]. As previously described, classical ECL emission only occurs when a potential is applied and shortly (µs) after the cessation of the applied potential due to characteristics of the system, such as the radical lifetime(s), excited state lifetimes, and the charge transfer kinetics [2, 17, 18, 34]. However, in “afterglow” chemiluminescence, there is a continual observation of luminescence intensity after the application of potential has stopped. Afterglow chemiluminescence phenomena have recently gained interest because they extend the luminescence lifetime, and hence the temporal resolution, from tens to hundreds of microseconds up to hundreds of seconds [15, 18, 19, 20]. In this case, as demonstrated by the ECL micrographs in Figure 3b, where the 0.000 s timepoint is the first frame of afterglow chemiluminescence (the first frame after the applied potential ceased), illumination at the spheroid–electrode interface via afterglow chemiluminescence continues for up to 180 s after the end of the applied potential.

It is important to distinguish the behavior we observed in this study from conventional long‐afterglow systems, in which emission persists due to the slow release of charge carriers from trap states within a material. In the present system, the afterglow arises from a fundamentally different mechanism governed by solution‐phase chemical processes. Specifically, emission persists due to the continued reaction of TPrA released from the spheroid with electrochemically generated [Ru(bpy)_3_]^3^^+^, enabling sustained production of the excited‐state [Ru(bpy)_3_]^2^^+^* in the absence of an applied potential. This “ECL‐initiated, coreactant‐release‐based afterglow chemiluminescence” therefore reflects a chemically driven process rather than a trap‐mediated electronic phenomenon.

To better understand this process and elucidate the origin of the sustained emission in our system, we consider the mechanistic pathways governing ECL generation under these conditions. The ECL emission and subsequent afterglow chemiluminescence are dominated by the catalytic pathway, as illustrated in Figure 3a. In this route, oxidation of TPrA to TPrA^•^^+^ is mediated by reaction with electrochemically generated [Ru(bpy)_3_]^3^^+^ rather than by direct electro‐oxidation at the electrode surface [42]. Following the formation of TPrA^•^^+^, the TPrA^•^^+^ undergoes deprotonation to generate TPrA^•^, which then reacts with another [Ru(bpy)_3_]^3^^+^ molecule to form the excited‐state [Ru(bpy)_3_]^2^^+^*. Hence, after the formation of TPrA^•+^, the reaction proceeds through the same sequence of steps as in the direct pathway to produce light.

This catalytic mechanism is enabled by the accumulation of [Ru(bpy)_3_]^3^^+^ in solution during the application of potential, as depicted in the dashed inset of Figure 3a. The buildup of this species provides a reservoir of oxidant that can subsequently react with TPrA released from the spheroid and confined near the spheroid–electrode interface. Notably, [Ru(bpy)_3_]^3^^+^ has previously been implicated in long‐lived afterglow chemiluminescence systems on the order of hundreds of seconds, supporting its role in sustaining emission in the present system [18]. Furthermore, while TPrA is locally enriched near the spheroid, it is likely present in excess relative to [Ru(bpy)_3_]^3^^+^, suggesting that the availability of [Ru(bpy)_3_]^3^^+^ is the limiting factor governing the duration of the afterglow. It is important to note that, while the direct oxidation pathway, in which TPrA is oxidized at the electrode before it reacts with [Ru(bpy)_3_]^3+^ to yield light, may also occur, its contribution is expected to be minor under these conditions due to the sluggish electron‐transfer kinetics of TPrA at ITO; accordingly, we detail this pathway in Figure S22.

This mechanistic framework is particularly important for understanding the origin of the afterglow chemiluminescence that we observe upon cessation of the applied potential (Figure 3b). Following removal of the bias, the continued release of TPrA from the spheroid can react with the relatively long‐lived, electrogenerated [Ru(bpy)_3_]^3^^+^, allowing the catalytic pathway to persist in the absence of an applied potential. This gives rise to sustained light emission for up to 180 s after the potential is removed, representing a unique example of ECL‐initiated, coreactant‐release‐driven afterglow chemiluminescence in a biological system. Finite‐element simulations further support this interpretation by capturing the spatial and temporal evolution of reactive intermediates during both the applied potential and afterglow regimes (Figure 3c,d). Figure 3c illustrates the concentration profile of TPrA^•^ during the application of potential for 180 s (images at 10, 60, and 180 s) as well as in the afterglow regime (images at 240 and 360 s). Figure 3c demonstrates that TPrA^•^ is generated not only at the electrode surface but also in regions extending into the solution and surrounding the spheroid, with the highest emission we observe still closest to the electrode surface. This spatial distribution is consistent with a catalytic mechanism in which [Ru(bpy)_3_]^3^^+^, generated at the electrode, diffuses away from the surface and oxidizes the TPrA that is released from the spheroid over time. In contrast, if the direct oxidation pathway were dominant, we would expect the TPrA^•^ formation to remain confined to the electrode surface.

We additionally support the temporal extension of the ECL signal with the finite‐element simulations of the concentration profiles of the excited‐state [Ru(bpy)_3_]^2^^+^* shown in Figure 3d, which remains detectable for up to 180 s after cessation of the applied potential. This sustained population of emissive species is consistent with the afterglow chemiluminescence that we experimentally observe (Figure 3b). It also indicates that the presence of long‐lived [Ru(bpy)_3_]^3^^+^ continues to drive the catalytic mechanism in the absence of an applied bias. Previously, our group has demonstrated that the electro‐oxidation of [Ru(bpy)_3_]^2+^ to [Ru(bpy)_3_]^3+^ in water can enable afterglow chemiluminescence lasting up to 10 min. Although this signal is considerably lower than what we observe in this study, the fact that it lasts up to 10 min supports that the lifetime of [Ru(bpy)_3_]^3+^ in water is at least that long. This validates that our 180‐s afterglow is, in part, driven by long‐lasting [Ru(bpy)_3_]^3+^ that can carry out the catalytic route to yield light. Together, our simulated concentration profiles of TPrA^•^ and [Ru(bpy)_3_]^2^^+^^*^ show persistent localization of reactive species near the spheroid interface, consistent with a slow release of TPrA and a diffusion‐limited propagation of the emission layer. These results support a mechanism in which localized accumulation and gradual release of TPrA sustain the ECL catalytic pathway, which governs both the intensity and persistence of the observed emission.

An alternative hypothesis might be that there is continued release of biological coreactants from the spheroid after the end of the voltage application period, which react with the oxidized [Ru(bpy)_3_]^3+^, which is produced in high concentrations at the electrode surface. To evaluate this, current versus time analysis was performed as before by holding the potential at 0.8 V for TPrA‐incubated spheroids and control spheroids, which were not exposed to TPrA during the reaction (Figure S23). Overall, we observed that while both cases resulted in afterglow, the spheroids that were incubated in TPrA exhibited afterglow for nearly 90 s post‐voltage shutoff, while the non‐incubated spheroids glowed for only about 30 s. Additionally, the TPrA‐based afterglow is of higher overall chemiluminescence intensity, specifically about 200 a.u. higher maximum afterglow chemiluminescence intensity than the non‐incubated spheroids, even though the current versus time traces between the two are comparable, which implies the conversion of [Ru(bpy)_3_]^2+^ to [Ru(bpy)_3_]^3+^ occurred at similar rates during the applied potential. This indicates that the biocoreactant has little impact on the ECL intensity and duration of the observed afterglow in this TPrA‐incubated spheroid system, and, hence, the TPrA acts as a much better coreactant for this afterglow chemiluminescence phenomenon. Additionally, it further supports the role of the electro‐oxidized [Ru(bpy)_3_]^3+^ in the afterglow mechanisms, as both the TPrA and biological coreactant exhibited afterglow ECL in the presence of [Ru(bpy)_3_]^3+^.

**FIGURE 4:**
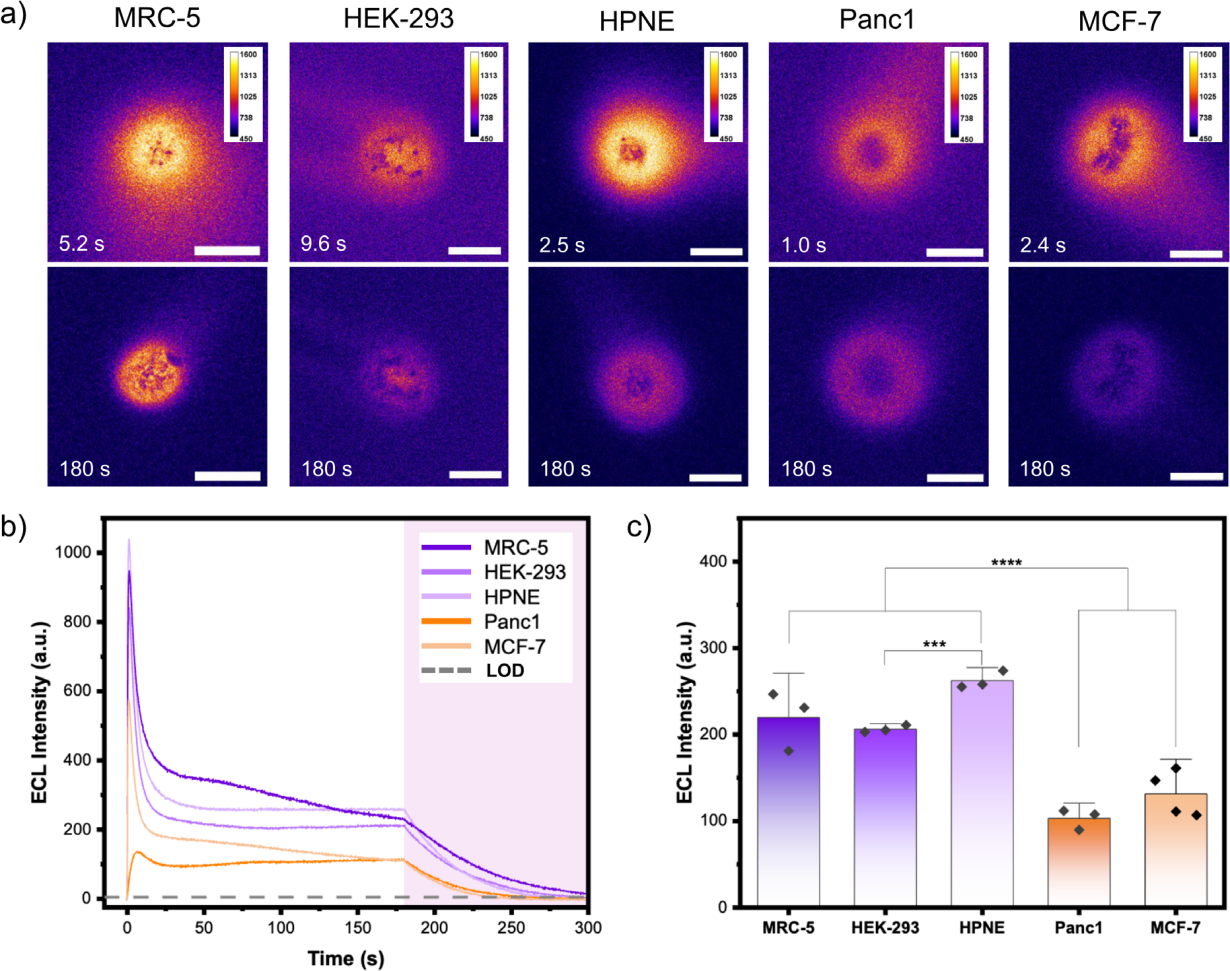
(a) ECL micrographs of spheroids from three non‐cancerous cell lines, MRC‐5, HEK‐293, and HPNE, and spheroids from two cancerous cell lines, Panc1 and MCF‐7. The top row includes an ECL micrograph for each type of spheroid within the first 10 s of the 180‐s voltage application of 1.1 V versus Ag wire. The bottom row shows an ECL micrograph from the last frame of the voltage application for each type of spheroid. All scale bars = 500 µm. (b) Intensity versus time plot where 1.1 V versus Ag wire was held for 180 s, then afterglow chemiluminescence was observed for about 120 s after the cessation of the applied potential. The afterglow chemiluminescence portion of the plot is highlighted in pink. The gray dashed line shows the LOD. (c) Comparison of ECL intensity in the last 15 frames of the applied potential of 1.1 V versus Ag wire before the cessation of potential. The spheroids derived from non‐cancerous cell lines, shown in shades of purple, as a group (*n* = 9), exhibit a statistically different ECL intensity in the last 15 frames of the 180‐s applied potential than the spheroids from cancerous cell lines (*n* = 7), shown in shades of orange, with a p value of < 0.0001. Within the group of spheroids from non‐cancerous cell lines, the spheroids from the HEK‐293 cell line (*n* = 3) also demonstrated a statistically different ECL intensity than the spheroids from the HPNE cell line (*n* = 3), with a p value of < 0.001. The spheroids from each non‐cancerous cell line showed statistically different ECL intensities than the spheroids from each cancerous cell line, which is summarized in Table S2.

With the Ru(bpy)_3_]^2+^‐TPrA ECL methodology thus established, we investigated its applicability across different cancerous and non‐cancerous cell lines to evaluate the difference in TPrA accumulation. Immortalized breast cancer (MCF‐7), and pancreatic cancer (Panc1) were used in addition to a non‐cancerous lung tissue cell line (MRC‐5), human embryonic kidney cells (HEK‐293), and Human Pancreatic Nestin‐Expressing (HPNE) cells. For this study, spheroids were generated using the MCF‐7 protocol described earlier, with the seeding number remaining consistent to enable direct comparison across cell lines. After spheroids reached maturation on Day 7, we conducted ECL analysis using constant potential application. Figure 4a shows the resulting ECL micrographs, with the first row of images showing the brightest frame we witnessed within the first 10 s of potential application for each cell line. The second row shows the intensity observed right before voltage cessation. The amperometric i‐t curves corresponding to Figure 4a are shown in Figure S24. As can be seen from Figure 4a, all cell lines yield spherical tumor models, but their ECL micrographs look quite different. Specifically, an important observation across these cell lines is the difference in shadowed areas in the ECL micrographs that arise from the points at which the spheroids attach to the electrode surface. Each cell line is unique in the extent to which its cells can interact with each other and their environment to form spheroids, as each cell line has been harvested from a different tissue in the body, each with its own distinct function [43, 44]. Therefore, the resulting spheroids from these cell lines also display variation in three‐dimensional topography and compactness. This gives rise to the distinct ECL micrographs seen for each cell line. For example, the MRC‐5 cell line shows relatively few and rather compact dark spots (Figure 4a). This is consistent with the tightly packed and nearly spherical spheroid that this cell line makes. Similarly, HEK‐293 shows a spread of single dark patches at the electrode surface (Figure 4a). These are more scattered compared to the previous spheroid‐type, indicating a loose attachment of cells. HPNE and Panc1 are both pancreatic cells and thus have similar morphologies (Figure 4a). They both show a sizable dark patch at the electrode surface right at the electrode | spheroid interface, indicating that these spheroids generally form low‐curvature spheroids. Lastly, the MCF‐7 cells showed a range of morphologies throughout the paper, indicating that the cell line does not have a preferred organization during spheroid formation. Overall, the differences in adhesion points only alter the firmness of the spheroid–electrode contact, but it does not visibly change the TPrA accumulation as seen in Figure 4b,c, where there seems to be no correlation between the intensity and adhesion points. This is likely because the overall surface area of the spheroid far exceeds the surface area of the spheroid‐electrode contact.

We also observed this disparity across cell lines, specifically between cancerous versus non‐cancerous cell lines, upon continuous potential application, such that the non‐cancerous cell lines consistently show a higher intensity compared to the cancerous cell lines. We compare this intensity difference in Figure 4b, which traces the intensity at the spheroid–electrode interface over time. While the cancerous cell lines show intensities of ∼100–200 a.u., the non‐cancerous cell line shows an average intensity of ∼200–400 a.u., a difference that is sustained throughout the 180 s of voltage application. Interestingly, in addition to disparities across the entire time that the voltage is applied, differences in the ECL intensity within the last 2 s of applied potential and at the beginning of the afterglow chemiluminescence are obvious between the spheroids from cancerous and non‐cancerous cell lines. Figure 4c compares peak intensity in the last 15 frames of ECL intensity (∼1.74 s) across three independent trials to show that the three non‐cancerous spheroids consistently yield higher ECL intensity as compared to the two cancerous cell lines. It is key to note that because these values are averaged across the last 15 frames, during which the intensity continues to evolve as the potential is continuously applied, the intensity values in this graph deviate from the visual seen in Figure 4a, where the ECL intensity is maximized because we captured the single image within the first 10 s of the application of potential. Furthermore, in the bottom row, the intensities that we report in Figure 4b,c also differ because they are average intensities across the whole spheroid, including any dark spots. Table S2 logs the statistical differences across all cell lines and highlights that each combination of cancerous to non‐cancerous cell line spheroids yields ECL intensities that are statistically different from each other. Importantly, we performed these comparisons in the pseudo‐steady‐state regime of the ECL response (Figure 4b), where we expect the signal to be dominated by TPrA release from within the spheroid rather than transient contributions from surface‐associated species. As such, the intensities we measured more directly reflect differences in coreactant accumulation and retention within the spheroid structure.

Furthermore, it is important to note that while the intensities differ, the length of afterglow chemiluminescence is consistent across the cell lines. This indicates that the extent of [Ru(bpy)_3_]^3+^ generation and lifetime at the electrode is consistent across the spheroids. Therefore, the afterglow chemiluminescence does not elucidate the cancerous or non‐cancerous identity of an individual cell line. However, the extent of TPrA accumulation differs across the samples, with the highest uptake occurring in the non‐cancerous cell line. While the precise origin of this difference remains to be fully established, it is likely rooted in variations in membrane composition and local microenvironment between cancerous and non‐cancerous cells [45]. Cancer cell membranes are known to undergo substantial lipid remodeling, including increased phospholipid content [46, 47], including phosphatidylcholine, phosphatidylethanolamine, and phosphatidylinositol; altered acyl chain composition; and reorganization into cholesterol‐ and sphingolipid‐rich lipid raft domains [48]. These changes are generally associated with increased membrane order and packing. Given that TPrA is relatively hydrophobic, its accumulation is expected to depend on its ability to partition into and be retained within lipid environments. Increased membrane order and raft enrichment in cancer cells may reduce bilayer permeability and limit TPrA insertion or retention, resulting in lower effective accumulation. In addition, the slightly acidic extracellular microenvironment often associated with cancer cells may influence the protonation state of TPrA, reducing its hydrophobicity and further hindering membrane partitioning.

Another possible explanation of the differences in the ECL intensities between spheroids from cancerous versus non‐cancerous cell lines that we observed could be that non‐cancerous cells are generally more susceptible to ROS stress induced by exogenously added reactants such as TPrA, and, thus, incubating the spheroids in the coreactants could upregulate the release of ROS species from the spheroid capable of independently interacting with the [Ru(bpy)_3_]^3+^ generation at the electrode. We show this in Figure S25, where we investigate an HPNE spheroid in 500 µM [Ru(bpy)_3_]^2+^ to monitor ECL in the absence of a coreactant. Based on the data, we saw a sizeable ECL intensity compared to baseline, even in the absence of an exogenously added coreactant. This is consistent with the results from MCF‐7 (Figures S7 and S23) we previously discussed. In both the HPNE and MCF‐7 spheroids, however, the signal was relatively weak, and the afterglow was about an order of magnitude shorter than when we incubated the spheroid in TPrA (tens vs. hundreds of seconds). We hypothesize that the ROS‐based ECL mechanisms are suppressed when the spheroid is incubated in TPrA, as the TPrA will directly compete with the ROS to react with the present [Ru(bpy)_3_]^3+^, and it may also slow both the diffusion of [Ru(bpy)_3_]^3+^ into the spheroid and the diffusion of ROS out of the spheroid. Hence, the [Ru(bpy)_3_]^2+^‐only ECL signal is likely minimized when the spheroid is incubated in TPrA. Therefore, the intensity we observed in all cell lines is most likely the amalgamation of both TPrA release and ROS release from inside the spheroid as a function of voltage‐induced stress.

To further investigate the origin of the observed differences in ECL intensity between spheroids derived from cancerous and non‐cancerous cell lines, we estimated the extent of TPrA accumulation within each system using a calibration‐based approach. First, we constructed a calibration curve relating ECL intensity to TPrA concentration by performing ECL measurements on bulk solutions containing 500 µM [Ru(bpy)_3_]^2^^+^, 25 mM glucose, and varying concentrations of TPrA (0–250 µM) in 1x DPBS (Figure S26). We conducted measurements in an ITO cell using an amperometric i–t experiment in which we applied 1.1 V versus Ag wire for 180 s. We recorded the ECL signals for the calibration curve under identical optical and electrochemical conditions as those used for spheroid measurements. For analysis, we averaged the final portion (last 15 frames) of the ECL signal during the applied potential to ensure consistency with spheroid‐based measurements, and we measured each concentration in triplicate. ECL intensity increases monotonically with TPrA concentration, enabling estimation of effective local coreactant concentrations within the spheroid environment. This relationship exhibits strong linearity (*R* ^2^ = 0.991), with a fitted slope of 3.9 a.u. µM^−^^1^, supporting its use for semi‐quantitative analysis.

We then applied this calibration to spheroids derived from five different human cell lines, including both cancerous and non‐cancerous systems. Following incubation in TPrA, we performed ECL measurements under identical conditions, and we converted the intensity values to estimated TPrA accumulation concentrations (Table S3). For this analysis, we only considered the final portion of the ECL signal during the applied potential, as this regime is expected to reflect TPrA released from within the spheroid rather than transient contributions from surface‐associated species. The results reveal clear and systematic differences in estimated TPrA accumulation across cell lines, with non‐cancerous spheroids exhibiting substantially higher effective TPrA concentrations relative to cancerous spheroids. Specifically, non‐cancerous cell lines such as HEK‐293, HPNE, and MRC‐5 show estimated TPrA concentrations in the range of approximately 50–70 µM, whereas cancerous cell lines such as MCF‐7 and Panc1 exhibit markedly lower values of approximately 25–35 µM (Table S3). This nearly twofold reduction in effective coreactant concentration directly mirrors the observed differences in ECL intensity and indicates that TPrA uptake and retention within the spheroid play a dominant role in defining the ECL response. Moreover, as an aside, we used these estimated spheroid TPrA uptake concentrations to guide the TPrA concentrations we used in the finite‐element simulations in Figure 2b and Figure S14.

It is important to note that these values represent effective concentrations that we derived from a calibration curve in a homogeneous solution and do not account for additional contributions to the ECL signal, such as ROS present within the spheroid microenvironment. As such, the values we report should be interpreted as semi‐quantitative estimates rather than absolute concentrations. Nevertheless, the consistent trends across multiple cell lines support the conclusion that differences in TPrA accumulation play a dominant role in governing the observed variation in ECL intensity.

## 3 Conclusion

In summary, we present the first application of the [Ru(bpy)_3_]^2+^–TPrA system for electrochemiluminescence imaging of multicellular spheroids. By leveraging TPrA pre‐incubation, we demonstrate that spheroids can accumulate and gradually release the coreactant, enabling sustained, high‐resolution ECL imaging without the need for exogenous labels. Importantly, this work also reveals a persistent afterglow chemiluminescence that continues well beyond the applied potential, allowing us to comment on the ability of the spheroid to accumulate enough TPrA such that in the presence of only [Ru(bpy)_3_]^3+^, ECL can continue at the spheroid interface because ECL is initiated by the bimolecular, homogeneous reaction between [Ru(bpy)_3_]^3+^ and TPrA. Moreover, this work demonstrated that striking differences in ECL emission intensity can be observed when comparing spheroids derived from cancerous versus non‐cancerous cell lines. Together, these findings establish the [Ru(bpy)_3_]^2+^–TPrA pair as a benchmark system for ECL studies in spheroids and open a path toward developing label‐free, high‐resolution ECL strategies for probing cancer biology. By setting this foundation, future efforts can harness the unique spatiotemporal capabilities of ECL to advance mechanistic understanding and therapeutic evaluation in three‐dimensional tumor models.

## Author Contributions

**Vanshika Gupta**: conceptualization, methodology, formal analysis, investigation, writing – original draft, writing – review and editing, visualization. **Megan L. Hill**: conceptualization, methodology, formal analysis, investigation, writing – original draft, writing – review and editing, visualization. **Samuel P. Nortz**: formal analysis, investigation, writing – review and editing, visualization. **Yashvi Choudhary**: investigation, visualization. **Ashutosh Rana**: formal analysis, investigation, writing – review and editing. **Jeffrey E. Dick**: conceptualization, methodology, writing – review and editing, supervision, funding acquisition.

## Conflicts of Interest

The authors declare that they (Vanshika Gupta, Megan L. Hill, Yashvi Choudhary, and Jeffrey E. Dick) have filed a provisional patent over this system and method.

## Supporting information




**Supporting File 1**: Supporting information for this manuscript, including cyclic voltammograms, fluorescence imaging, and live‐dead spheroid analysis.

**Supporting File 2**: Corresponding videos for selected ECL experiments shown in the main text.

## Data Availability

The data that support the findings of this study are available from the corresponding author upon reasonable request.
