# Supplementary material for: Localized Accumulation of Tri‐n‐Propylamine Prolongs Electrochemiluminescence for Tumor Model Spheroid Analysis
Source: Angew Chem Int Ed Engl. 2026 Jun 11;65(33):e8508105. doi: 10.1002/anie.8508105 (PMC13452574; doi:10.1002/anie.8508105)
Supplement: Supplementary file 1 [file ANIE-65-e8508105-s002.docx]

*Supporting Information for:*

**Localized Accumulation of Tri-n-propylamine Prolongs Electrochemiluminescence for Tumor Model Spheroid Analysis**

Vanshika Gupta ^a+^, Megan L. Hill^a+^, Samuel P. Nortz^a^, Yashvi Choudhary^a^, Ashutosh Rana^a^, and Jeffrey E. Dick^a,b^*

^a^ Department of Chemistry, Purdue University, West Lafayette, IN 47906, USA

^b^ Elmore Family School of Electrical and Computer Engineering, Purdue University, West Lafayette, IN 47906, USA

*To whom correspondence should be addressed: [jdick@purdue.edu](mailto:jdick@purdue.edu)

^+^Indicates equal contribution

**Table of Contents**

| **Description** | **Page(s)** |
| --- | --- |
| **Materials and Methods** | S3-S4 |
| **Figure S1.** Experimental setup of the ECL microscopy experiments. | S5 |
| **Figure S2.** 500 µM [Ru(bpy)_3_]^2+^ and 250 µM TPrA ECL imaging of an MCF-7 spheroid. | S6 |
| **Figure S3.** ECL imaging of an MCF-7 spheroid with an incubation in 500 µM [Ru(bpy)_3_]^2+^. | S7 |
| **Supplemental Note 1.** Experimental details on how the applied bias was selected for the amperometric i-t curves throughout the study. | S8 |
| **Figure S4.** Cyclic voltammograms of a [Ru(bpy)_3_]^2+^ only solution with both Ag wire quasi-reference electrodes used throughout this study. | S9 |
| **Figure S5.** Brightfield, Fluorescence, and ECL Microscopy of spheroids from the HPNE cell line in different incubation solutions. | S10 |
| **Figure S6.** Brightfield, Fluorescence, and ECL Microscopy of spheroids from the Panc1 cell line in different incubation solutions. | S11 |
| **Figure S7.** ECL imaging of an MCF-7 spheroid in 500 µM [Ru(bpy)_3_]^2+^ only. | S12 |
| **Figure S8.** ECL imaging of an MCF-7 spheroid in 250 µM TPrA only. | S13 |
| **Figure S9.** Fluorescence assay with increasing concentration of TPrA. | S14 |
| **Figure S10.** Different TPrA Concentrations. | S15 |
| **Figure S11.** Comparison of ECL intensity vs time and the CVs for an incubation time of 5 minutes versus 15 minutes for the spheroid in 250 µM TPrA. | S16 |
| **Supplemental Note 2.** Details on the COMSOL Simulations. | S17 |
| **Table S1.** Parameters used in the COMSOL finite-element simulations. | S18 |
| **Figure S12.** The mesh setting for the COMSOL simulations. | S19 |
| **Figure S13**. Finite-element simulation of differing TPrA release kinetics from a spheroid at 180 s into the application of potential. | S20 |
| **Figure S14**. Finite-element simulation of the ECL signal over time with different amounts of TPrA accumulation within the spheroid. | S21 |
| **Figure S15.** CVs that correspond to the points in the ECL intensity versus time plot in Figure 2d and the ECL micrographs in Figure 2c. | S22 |
| **Figure S16**. TPrA leakage investigation based on the ECL intensity with a spheroid in solution (pre-extraction) and after the spheroid is removed (post-extraction). | S23 |
| **Figure S17.** ECL microscopy in a [Ru(bpy)_3_]^2+^-only solution without a spheroid. | S24 |
| **Figure S18.** Serial trypsinization of spheroid to visualize TPrA accumulation. | S25 |
| **Figure S19.** CVs, ECL intensity vs applied potential plots, ECL micrographs, and comparison of the maximum ECL intensity for 125 µM, 250 µM, 500 µM, and 750 µM [Ru(bpy)_3_]^2+^ solutions. | S26 |
| **Figure S20.** Amperometric it-curves, ECL intensity vs time plots, ECL micrographs, and comparison of the maximum ECL intensity and afterglow durations for 125 µM, 250 µM, 500 µM, and 750 µM [Ru(bpy)_3_]^2+^ solutions. | S27 |
| **Figure S21.** Amperometric i-t curve corresponding to Figure 3. | S28 |
| **Figure S22.** Direct oxidation pathway for [Ru(bpy)_3_]^2+^ / TPrA ECL. | S29 |
| **Figure S23.** Afterglow ECL intensity vs time plot and amperometric it-curve comparing an MCF-7 spheroid with the TPrA incubation versus a non-incubated MCF-7 spheroid. | S30 |
| **Figure S24.** Amperometric i-t curves corresponding to the ECL micrographs in Figure 4a. | S31 |
| **Table S2.** Comparison of the ECL intensity from the last 15 frames of applied potential between spheroids from each type of non-cancerous to cancerous cell lines. | S32 |
| **Figure S25.** ECL Microscopy of a spheroid from the HPNE cell line without incubation in TPrA. | S33 |
| **Figure S26.** Calibration curve of ECL intensity (a.u.) vs TPrA concentration (µM). | S34 |
| **Table S3.** Estimated TPrA accumulation in spheroids derived from both cancerous and non-cancerous human cell lines based on their ECL intensities. | S35 |
| **Other supplementary materials for this manuscript include the following:** |  |
| **Movies S1-S10** | S36-S37 |

**Materials and Methods**

*Materials and reagents*

All materials and reagents were used as received unless otherwise specified. Indium tin oxide (ITO, 100 mm x 100 mm x 1.1 mm) electrodes were sourced from Qunguan Electronics, China, and a glassy carbon rod (d = 2 mm, L = 10 cm) counter electrode was obtained from CH Instruments (Austin, Tx). Silver (Ag) wire quasi-reference electrodes, tri-n-propylamine (TPrA, 98%), and Dulbecco’s phosphate-buffered saline (DPBS, 1X, without calcium or magnesium) were purchased from Sigma-Aldrich. Dextrose (99.5%) and tris(2,2'-bipyridyl)ruthenium(II) chloride hexahydrate ([Ru(bpy)_3_][Cl_2_]·6H_2_O, 98%) were obtained from Thermo Fisher Scientific.

*Spheroid formation*

All immortalized human cell lines were procured from ATCC. Estrogen receptor-positive breast cancer cells (MCF-7; HTB-22), ductal pancreatic cancer cells (PANC-1; CRL-1469), epithelial noncancerous pancreatic cells ( hTERT-HPNE; CRL-4023), embryonic kidney cells (HEK-293; CEL-1573), and lung fibroblast cells (MRC5-5; CCL-171) were cultured in Dulbecco’s Modified Eagle’s Medium (DMEM, high glucose; Sigma-Aldrich) supplemented with 10% fetal bovine serum (VWR) and 1.5% penicillin-streptomycin (Sigma-Aldrich). Cells were maintained at 37 °C in a humidified incubator with 5% CO₂ until approximately 90% confluence. At this point, cells were detached using TrypLE™ Express Enzyme (Thermo Fisher Scientific) and seeded into ultra-low attachment, round-bottom 96-well plates (Corning) at a density of 1 × 10⁵ cells/mL (1 × 10^4^ cells/well). All spheroids were cultured for 7 days prior to experimentation, with 75% of the culture medium replaced every other day before use in experiments.

*Electrochemiluminescence microscopy*

Electrochemiluminescence (ECL) microscopy was performed using a Nikon Eclipse Ti2 inverted microscope (Nikon, Japan). Fluorescence imaging utilized a D-LEDi light source, while brightfield imaging employed an external MKII light source (Nikon, Japan). A 4× objective lens (numerical aperture: 0.20, working distance: 20 mm) was used throughout, with the internal 1.0× optical magnifier engaged for a total magnification of 4×. Images were captured using an EMCCD iXon 888 camera (Andor Technology Ltd., Belfast, UK) with a 400 EM gain, 116 ms exposure time (unless otherwise stated), operated in 10 MHz quality mode, using Gain 2, a shift speed of 4.22 μs, and maintained between -75°C to -85°C. Image acquisition was controlled via NIS Elements software (Nikon, Japan). Electrochemical control was provided by a CH Instruments 601E potentiostat (CH Instruments, Austin, TX, USA). A Hamamatsu ORCA-Quest CMOS C15550-20UP camera (Hamamatsu, Japan) was also available for complementary imaging.

*Electrochemical Experiments*

Electrochemical cells were assembled in-house by adhering a round glass cylinder (i internal diameter: 19 mm, height: 20 mm, Scientific Glass Blowing Lab, Purdue University Department of Chemistry) onto an ITO working electrode using epoxy (Gorillatough.com, USA), cured overnight. Prior to each experiment, the integrity of the seal was verified. Fresh culture medium and a single 7-day-old spheroid were added to each cell. The medium was then replaced with 250 µM TPrA in 1x DPBS supplemented with 25 mM glucose, and the spheroid was incubated for 15 minutes. Following incubation, the TPrA solution was removed without disturbing the spheroid, and replaced with 500 µM Rubpy solution prepared in the same DPBS/glucose mixture. The ITO working electrode was connected to a CH Instruments 601E potentiostat via copper tape, with a silver (Ag) wire quasi-reference electrode and a carbon rod counter electrode completing the electrochemical setup. For the amperometric it-curve experiments, either 0.8 V or 1.1 V vs Ag wire was held for 180 seconds (depending on which Ag wire was used), while for the cyclic voltammogram (CV) experiments, the CVs were swept from 0 to 1.3 V vs Ag wire at a scan rate of 50 mV/s.

**Figure S1.** **Experimental setup of the ECL microscopy experiments.** In these experiments, an optically transparent ITO electrode was the working electrode, a glassy carbon rod was the counter electrode, and an Ag wire served as a quasi-reference electrode. The 4x objective was focused on the spheroid | ITO electrode interface, the brightfield imaging was performed with a CMOS camera, and the ECL microscopy was performed with the EMCCD camera. The spheroid was immersed in a 500 µM [Ru(bpy)_3_]^2+^ and 25 mM glucose within 1x DPBS.

**Figure S2.** **500 µM [Ru(bpy)_3_]^2+^ and 250 µM TPrA ECL imaging of an MCF-7 spheroid. A)** Schematic showing the non-incubated experimental setup wherein ECL was performed on an MCF-7 spheroid within a 500 µM [Ru(bpy)_3_]^2+^, 250 µM TPrA, and 4500 mg/L dextrose in a 1x DPBS solution. Brightfield micrograph (**B**), ECL micrograph (**C**), and ECL intensity vs time plot (**D**) of the MCF-7 spheroid. **E)** Amperometric it-curve of the MCF-7 spheroid, where a potential of 0.8 V vs Ag wire was applied for 180 seconds. The LOD was determined by taking the standard deviation of the first 15 frames, before the application of potential, and multiplying it by a factor of three. The ECL intensity reported in these plots was also background-subtracted, where the background was set to be the average of the first 15 frames of the video (the same frames used for the calculation of the LOD). All scale bars = 500 µm and all the images were collected with a 500 ms exposure.

**Figure S3.** **ECL imaging of an MCF-7 spheroid following an incubation in 500 µM [Ru(bpy)_3_]^2+^.** An MCF-7 spheroid was incubated in 500 µM [Ru(bpy)_3_]^2+^ and 4500 mg/L dextrose in a 1x DPBS solution for 15 minutes, then ECL was performed in a 250 µM TPrA and 4500 mg/L dextrose in a 1x DPBS solution. Brightfield micrograph (**A**), ECL micrograph (**B**), and ECL intensity vs time plot (**C**) of the MCF-7 spheroid. **D)** Amperometric it-curve of the MCF-7 spheroid, where a potential of 0.8 V vs Ag wire was applied for 180 seconds. The LOD was determined by taking the standard deviation of the first 15 frames, before the application of potential, and multiplying it by a factor of three. The ECL intensity reported in these plots was also background-subtracted, where the background was set to be the average of the first 15 frames of the video (the same frames used for the calculation of the LOD). All scale bars = 500 µm and all the images were collected with a 500 ms exposure.

**Supplemental Note 1. Experimental details on how the applied bias was selected for the amperometric i-t curves throughout the study.**

Throughout this study, two different Ag wires were used as the quasi-reference electrode. Quasi-reference electrodes (QREs) differ from typical reference electrodes used within electrochemistry because they do not have a stable and well-known electrode potential. Rather, QREs can exhibit a reproducible and stable reference potential over the course of a series of measurements, but their potential often varies from system to system.^1,2^ Hence, as two different QREs were used during the study, they differed in the potential at which the oxidation of [Ru(bpy)_3_]^2+^ to [Ru(bpy)_3_]^3+^ occurred. **Figure S4** depicts cyclic voltammograms (CVs) in the IUPAC convention of a bulk solution of 500 µM [Ru(bpy)_3_]^2+^ and 25 mM glucose in 1x DPBS within an ITO cell using QRE 1 (blue) as opposed to QRE 2 (red). Both CVs were taken in a potential window of 0 to 1.3 V at a scan rate of 50 mV/s and had a glassy carbon rod counter electrode. As the CVs were taken on different ITO cells, which have a variable electrode size, the CVs show different anodic and cathodic peak currents because of the different electrochemically active surface areas exposed to the solution. Given that the anodic and cathodic peak potentials are shifted from QRE 1 to QRE 2 by ~ 300 mV for the [Ru(bpy)_3_]^2+^ / [Ru(bpy)_3_]^3+^ pair, different applied potentials were necessary when using these QREs to bulk oxidize [Ru(bpy)_3_]^2+^ using an amperometric i-t curve within the study. Hence, based on **Figure S4**, a potential of 0.8 V was held for QRE 1, which is a 50 mV overpotential from the anodic peak potential of ~0.75 V. Whereas for QRE 2, a potential of 1.1 V was used for the amperometric i-t curves, which is a 50 mV overpotential from the anodic peak potential of ~1.05 V. Before each experiment, a CV was taken in a bulk solution of 500 µM [Ru(bpy)_3_]^2+^ and 25 mM glucose in 1x DPBS to validate that the anodic peak potential has not shifted from 0.75 V and 1.05 V for QRE 1 and QRE 2, respectively.

References:

1. Bard, A.J., Faulkner, L.R., and White, H.S. (2022) *Electrochemical methods: Fundamentals and applications*, John Wiley & Sons, Ltd, Hoboken, NJ.

2. Lee, J., Jäckel, N., Kim, D., et al. (2016) Porous carbon as a quasi-reference electrode in aqueous electrolytes. *Electrochimica Acta*, **222**, 1800–1805.

**Figure S4. Cyclic Voltammograms of a [Ru(bpy)_3_]^2+^ only solution with both Ag wire quasi-reference electrodes used throughout this study.** The cyclic voltammograms (CVs) in the IUPAC convention of a bulk solution of 500 µM [Ru(bpy)_3_]^2+^ and 25 mM glucose in 1x DPBS within an ITO cell using QRE 1 (blue) as opposed to QRE 2 (red). Both CVs were taken in a potential window of 0 to 1.3 V at a scan rate of 50 mV/s and had a glassy carbon rod counter electrode. The oxidation of [Ru(bpy)_3_]^2+^ to [Ru(bpy)_3_]^3+^ reaches an anodic peak potential at ~0.75 V for QRE 1 and ~1.05 for QRE 2. For further discussion of this figure, refer to **Supplemental Note 1**.

**Figure S5. Brightfield, Fluorescence, and ECL Microscopy of spheroids from the HPNE cell line in different incubation solutions.** Brightfield microscopy **(A)**, fluorescence microscopy **(B)**, and ECL microscopy **(C)** of a spheroid after a 15-minute incubation in 500 µM [Ru(bpy)_3_]^2+^ and 25 mM glucose in 1x DPBS. The ECL microscopy was performed in a solution of 250 µM TPrA and 25 mM glucose in 1x DPBS. Brightfield microscopy **(D)**, fluorescence microscopy **(E)**, and ECL microscopy **(F)** of a spheroid after a 15-minute incubation in 250 µM TPrA and 25 mM glucose in 1x DPBS. The ECL microscopy was performed in a solution of 500 µM [Ru(bpy)_3_]^2+^ and 25 mM glucose in 1x DPBS. Brightfield microscopy **(G)**, fluorescence microscopy **(H)**, and ECL microscopy **(I)** of a spheroid after a 15-minute incubation in 500 µM [Ru(bpy)_3_]^2+^, 250 µM TPrA, and 25 mM glucose in 1x DPBS. The ECL microscopy was performed in a solution of 1x DPBS. For each incubation condition, the fluorescence microscopy was carried out in 1x DPBS, using a 550 nm excitation light, and with a bandpass filter (604-679 nm) to limit the accepted wavelengths to the EMCCD detector to the expected *ca.* 620 nm emission wavelength of [Ru(bpy)_3_]^2+^. Additionally, all ECL microscopy was carried out by using an amperometric it-curve held at 1.1 V vs Ag wire for 180 seconds. The experimental set-up shown in *Figure S1* was used for the ECL microscopy.

**Figure S6. Brightfield, Fluorescence, and ECL Microscopy of spheroids from the Panc1 cell line in different incubation solutions**. Brightfield microscopy **(A)**, fluorescence microscopy **(B)**, and ECL microscopy **(C)** of a spheroid after a 15-minute incubation in 500 µM [Ru(bpy)_3_]^2+^ and 25 mM glucose in 1x DPBS. The ECL microscopy was performed in a solution of 250 µM TPrA and 25 mM glucose in 1x DPBS. Brightfield microscopy **(D)**, fluorescence microscopy **(E)**, and ECL microscopy **(F)** of a spheroid after a 15-minute incubation in 250 µM TPrA and 25 mM glucose in 1x DPBS. The ECL microscopy was performed in a solution of 500 µM [Ru(bpy)_3_]^2+^ and 25 mM glucose in 1x DPBS. Brightfield microscopy **(G)**, fluorescence microscopy **(H)**, and ECL microscopy **(I)** of a spheroid after a 15-minute incubation in 500 µM [Ru(bpy)_3_]^2+^, 250 µM TPrA, and 25 mM glucose in 1x DPBS. The ECL microscopy was carried out in a solution of 1x DPBS. For each incubation condition, the fluorescence microscopy was performed in 1x DPBS, using a 550 nm excitation light, and with a bandpass filter (604-679 nm) to limit the accepted wavelengths to the EMCCD detector to the expected *ca.* 620 nm emission wavelength of [Ru(bpy)_3_]^2+^. Additionally, all ECL microscopy was conducted by using an amperometric it-curve held at 1.1 V vs Ag wire for 180 seconds. The experimental set-up depicted in *Figure S1* was used for the ECL microscopy.

**Figure S7.** **ECL imaging of an MCF-7 spheroid in 500 µM [Ru(bpy)_3_]^2+^ only.** An MCF-7 spheroid was interrogated via ECL microscopy within a 500 µM [Ru(bpy)_3_]^2+^ and 4500 mg/L dextrose in a 1x DPBS solution. Brightfield micrograph (**A**), ECL micrograph (**B**), and ECL intensity vs time plot (**C**) of the MCF-7 spheroid. **D)** Amperometric it-curve of the MCF-7 spheroid, where a potential of 0.8 V vs Ag wire was applied for 180 seconds. The LOD was determined by taking the standard deviation of the first 15 frames, before the application of potential, and multiplying it by a factor of three. The ECL intensity reported in these plots was also background-subtracted, where the background was set to be the average of the first 15 frames of the video (the same frames used for the calculation of the LOD). All scale bars = 500 µm and all the images were collected with a 500 ms exposure.

**Figure S8.** **ECL imaging of an MCF-7 spheroid in 250 µM TPrA only.** An MCF-7 spheroid was interrogated via ECL microscopy within a 250 µM TPrA and 4500 mg/L dextrose in a 1x DPBS solution. Brightfield micrograph (**A**), ECL micrograph (**B**), and ECL intensity vs time plot (**C**) of the MCF-7 spheroid. **D)** Amperometric it-curve of the MCF-7 spheroid, where a potential of 0.8 V vs Ag wire was applied for 180 seconds. The LOD was determined by taking the standard deviation of the first 15 frames, before the application of potential, and multiplying it by a factor of three. The ECL intensity reported in these plots was also background-subtracted, where the background was set to be the average of the first 15 frames of the video (the same frames used for the calculation of the LOD). All scale bars = 500 µm and all the images were collected with a 500 ms exposure.

**Figure S9.** **Fluorescence assay with increasing concentration of TPrA.** Blue fluorescence signal refers to the Hoechst stain which interacts with the DNA in all cells. Red fluorescence signal is obtained when the dye Propidium Iodide interacts enters the membrane of dead cells and chelates into their DNA.

**Figure S10.** **Different TPrA Concentrations.** In these experiments, an optically transparent ITO electrode was the working electrode, a glassy carbon rod was the counter electrode, and an Ag wire served as a quasi-reference electrode. Moreover, the spheroid was incubated in varying concentrations of TPrA: **A)** 125 µM, **B)** 250 µM, **C)** 500 µM, and **D)** 750 µM with 4500 mg/L dextrose in a 1x DPBS solution for 15 minutes, then ECL was performed in a 500 µM [Ru(bpy)_3_]^2+^ and 4500 mg/L dextrose in a 1x DPBS solution. **E)** The CVs were swept from 0 to 1.3 V vs Ag wire at a scan rate of 50 mV/s.

**Figure S11.** **Comparison of ECL intensity vs time and the CVs for an incubation time of 5 minutes versus 15 minutes for the spheroid in 250 µM TPrA.** **A.** ECL intensity vs time plot and **B.** CVs of a spheroid that had a 5-minute TPrA incubation vs a spheroid that had a 15-minute TPrA incubation within a 500 µM [Ru(bpy_)3_]^2+^ with 4500 mg/L dextrose in a 1x DPBS solution. The 15-minute incubation time was chosen as it had a higher ECL intensity compared to the 5-minute incubation. The LOD was determined as described in the figure caption for *Figure S2*.

**Supplemental Note 2. Details on the COMSOL Simulations.**

COMSOL Multiphysics 6.1.282 was used to build finite element models of the tri-n-propylamine localization and ECL reaction mechanism at a spheroid | electrode interface. This provides insight into the electrogenerated and afterglow ECL mechanism, as well as coreactant depletion and mass-transfer hinderance at the spheroid | electrode interface. All COMSOL simulation parameters shown in **Table S1** and bimolecular chemical rate equations were adopted from Ding et al^1^. The geometry and meshing used in the simulation are shown in **Figure S12**.

References:

1. Ding, H.; Zhou, P.; Fu, W.; Ding, L.; Guo, W.; Su, B. Spatially Selective Imaging of Cell–Matrix and Cell–Cell Junctions by Electrochemiluminescence. *Angewandte Chemie - International Edition* 2021, *60* (21). https://doi.org/10.1002/anie.202101467.

**Table S1. Parameters used in the COMSOL finite-element simulations.**

| **Name** | **Value** | **Description** |
| --- | --- | --- |
| C_Rubpy | 0.5 mol/m³ | Initial [Ru(bpy)_3_]^2+^ Concentration |
| C_TPrA | 0.03, 0.06, and 0.120 mol/m³ | Initial TPrA Concentration |
| D_Ru | 5.9 x 10^−10^ m²/s | Diffusion coefficient of [Ru(bpy)_3_]^2+^, [Ru(bpy)_3_]^3+^, and [Ru(bpy)_3_]^2+*^ |
| D_TPrA | 5 x 10^−11^, 5 x 10^−13^, and  5 x 10^−15^ m²/s | Diffusion coefficient of TPrA, TPrA^•^, and TPrA^•+^ |
| L_bulk | 0.001 m | Length of bulk solution boundary |
| F | 96485 C/mol | Faraday's constant |
| k1 | 0.15 m/s | Rate constant of [Ru(bpy)_3_]^2+^ oxidation to [Ru(bpy)_3_]^3+^ |
| alpha | 0.5 | Transfer coefficient |
| R_g | 8.3145 J/(mol·K) | Gas Constant |
| T | 298 K | Temperature |
| f | 38.941 1/V | f defined in Butler Volmer |
| overpot | 0.1 V | Overpotential |
| k2 | 1E−4 m/s | Rate constant of TPrA oxidation to TPrA^•+^ |
| k3 | 3500 1/s | Rate constrant of TPrA^•+^ deprotonation to TPrA^•^ |
| k4 | 1E−4 m/s | Rate constant of TPrA^•^ oxidation to P_0_ |
| k5 | 13 m³/(s·mol) | Rate constant of the homogeneous catalytic step |
| k6 | 1000 m³/(s·mol) | Rate constant of [Ru(bpy)_3_]^2+^ excitation to [Ru(bpy)_3_]^2+*^ |
| k7 | 300 1/s | Rate constant of the radiative relaxation |
| t_tot | 180 s | Time potential applied |

*All [Ru(bpy)_3_]^2+^ and TPrA rate constants and diffusion coefficients were adopted from Ding et al. Ref [10]

**Figure S12. The mesh setting for the COMSOL simulations**. This meshing was used to simulate the transport of the dilute TPrA and [Ru(bpy)_3_]^2+*^ species around a spherical geometry, representing a spheroid at the surface of an electrode. Specifically, as shown in this meshing, a thin-layer boundary condition was used outside the spheroid to mimic the localization and preferential binding of TPrA to the outer spheroid surface.

**Figure S13**. **Finite-element simulation of differing TPrA release kinetics from a spheroid at 180 s into the application of potential.** In this simulation, differing TPrA release kinetics from a spheroid were evaluated by using a thin-layer boundary condition outside the spheroid with a variable diffusion coefficient to mimic differing release kinetics. The diffusion coefficients interrogated were: *D_TPrA_* = 5x10^-11^ m^2^/s (top row), *D_TPrA_* = 5x10^-13^ m^2^/s (middle row), and *D_TPrA_* = 5x10^-15^ m^2^/s (bottom row). A potential hold with a 0.1 V overpotential for the oxidation of [Ru(bpy)_3_]^2+^ to [Ru(bpy)_3_]^3+^ for 180 s was included in the simulation, with the 360 s time point (far right column) representing afterglow chemiluminescence. The ECL intensity is shown with the calibration curve as a function of [Ru(bpy)_3_]^2+*^ (excited state [Ru(bpy)_3_]^2+^), with the higher the concentration of [Ru(bpy)_3_]^2+*^, the higher the ECL signal.

**Figure S14**. **Finite-element simulation of the ECL signal over time with different amounts of TPrA accumulation within the spheroid.** In this simulation, three different concentrations of TPrA were evaluated: 30 µM (top row), 60 µM (middle row), and 120 µM (bottom row). This concentration of TPrA was modeled to be in the region immediately surrounding the spheroid, represented by the black circle, with a *D_TPrA_* = 5x10^-11^ m^2^/s assumed for each TPrA concentration. A potential hold with a 0.1 V overpotential for the oxidation of [Ru(bpy)_3_]^2+^ to [Ru(bpy)_3_]^3+^ for 180 s was included in the simulation, with the 300 s time point (far right column) representing afterglow chemiluminescence. The ECL intensity is shown with the calibration curve as a function of [Ru(bpy)_3_]^2+*^ (excited state [Ru(bpy)_3_]^2+^), with the higher the concentration of [Ru(bpy)_3_]^2+*^, the higher the ECL signal.

**Figure S15.** **CVs that correspond to the points in the ECL intensity versus time plot in Figure 2d and the ECL micrographs in Figure 2c.** In these experiments, an optically transparent ITO electrode was the working electrode, a glassy carbon rod was the counter electrode, and an Ag wire served as a quasi-reference electrode. Moreover, the spheroid was incubated in 250 µM TPrA and 25 mM glucose in 1x DPBS solution for 15 minutes, and then ECL was performed in a 500 µM [Ru(bpy)_3_]^2+^ and 25 mM glucose in 1x DPBS solution. The CVs were swept from 0 to 1.3 V vs Ag wire at a scan rate of 50 mV/s.

**Figure S16.** **TPrA leakage investigation based on the ECL intensity with a spheroid in solution (pre-extraction) and after the spheroid is removed (post-extraction).** In the pre-extraction, the spheroid was incubated in 250 µM TPrA and 4500 mg/L dextrose in a 1x DPBS solution for 15 minutes, and then ECL was performed in a 500 µM [Ru(bpy)_3_]^2+^ and 4500 mg/L dextrose in a 1x DPBS solution. **A.** ECL micrograph of the spheroid. **B.** ECL intensity vs time plot of the spheroid shown in A. The LOD was determined as described in the figure caption for *Figure S2*. **C.** CV carried out during the experiment that corresponds to A and B, the CV was swept from 0 to 1.3 V vs Ag wire at a scan rate of 50 mV/s. In the post-extraction, the spheroid was left in the [Ru(bpy)_3_]^2+^ solution for 30 minutes, then removed, and the ECL was run again. **D.** ECL micrograph of the leftover solution without the spheroid. **E.** ECL intensity vs time plot of the leftover solution. **C.** CV carried out during the experiment that corresponds to D and E, the CV was swept from 0 to 1.3 V vs Ag wire at a scan rate of 50 mV/s.

**Figure S17. ECL microscopy in a [Ru(bpy)_3_]^2+^-only solution without a spheroid**. **A)** ECL intensity vs applied potential for a bulk solution of 500 µM [Ru(bpy)_3_]^2+^ and 25 mM glucose in 1x DPBS using the same experimental setup as shown in *Figure S1*. The ECL was driven by a cyclic voltammogram swept from 0 to 1.3 V vs Ag wire at a scan rate of 50 mV/s. **B)** ECL intensity vs time for a bulk solution of 500 µM [Ru(bpy)_3_]^2+^ and 25 mM glucose in 1x DPBS using the same experimental setup as shown in *Figure S1*. The ECL was driven by an amperometric it curve held at 1.1 V vs Ag wire for 180 seconds. **C)** Cyclic voltammograms corresponding to the trial shown in (A). **D)** Amperometric i-t curve corresponding to the trial shown in (B). For both (A) and (B), the plotted ECL intensities were background-subtracted by taking the average intensity of 15 frames before performing ECL, then subtracting it from the measured intensities. Moreover, the limit of detection (LOD) in both (A) and (B) was determined by taking the standard deviation of 15 frames prior to ECL and multiplying this standard deviation by 3.

**Figure S18.** **Serial trypsinization of spheroid to visualize TPrA accumulation.** In this experiment, spheroids were incubated as before and the voltage was swept using cyclic voltammetry while the resulting ECL was recorded. The spheroid was then harvested and the first few layers of cells were disrupted using the enzyme trypsin. The resulting cells and spheroids were isolated and re-interrogated in a fresh solution of [Ru(bpy)_3_]^2+^. **A.** Brightfield image of spheroid. **B.** ECL image of spheroid. **C.** ECL intensity resulting from the potentials sweep. **D.** Bright field of the harvested spheroids, post-trypsinization. **E.** ECL of the harvested spheroids, post-trypsinization. **F.** ECL intensity over time as the potential is kept at 0.8 V of harvested spheroids. **G.** Brightfield of the harvested cells, post-trypsinization. **H.** ECL of the harvested cells, post-trypsinization. **I.** ECL intensity over time as the potential is kept at 0.8 V of harvested cells.

**Figure S19.** **CVs, ECL intensity vs applied potential plots, ECL micrographs, and comparison of the maximum ECL intensity for 125 µM, 250 µM, 500 µM, and 750 µM [Ru(bpy)_3_]^2+^ solutions.** **A.** Overlay plot of the CVs for the different concentrations of [Ru(bpy)_3_]^2+^, each swept from 0 to 1.3 V vs Ag wire. **B.** ECL intensity vs applied potential plots for each [Ru(bpy)_3_]^2+^ concentration. **C.** Maximum ECL intensity vs [Ru(bpy)_3_]^2+^ concentration plot. For both B and C, the LOD was determined as described in the figure caption for *Figure S2*. ECL micrographs for 125 µM [Ru(bpy)_3_]^2+^ (**Di**), 250 µM [Ru(bpy)_3_]^2+^ (**Dii**), 500 µM [Ru(bpy)_3_]^2+^ (**Diii**), and 750 µM [Ru(bpy)_3_]^2+^ (**Div**) solutions. In these experiments, the spheroids were first incubated in 250 µM TPrA and 4500 mg/L dextrose in a 1x DPBS solution for 15 minutes, then ECL was carried out in the varied concentration of [Ru(bpy)_3_]^2+^ and 4500 mg/L dextrose in 1x DPBS solutions.

**Figure S20. Amperometric it-curves, ECL intensity vs time plots, ECL micrographs, and comparison of the maximum ECL intensity and afterglow durations for 125 µM, 250 µM, 500 µM, and 750 µM [Ru(bpy)_3_]^2+^ solutions.** **A.** Overlay plot of the amperometric it-curves for the different concentrations of [Ru(bpy)_3_]^2+^, each held at 0.8 V vs Ag wire for 180 seconds. **B.** ECL intensity vs time plots for each [Ru(bpy)_3_]^2+^ concentration. **C.** Maximum ECL intensity and afterglow time vs [Ru(bpy)_3_]^2+^ concentration plot. For both B and C, the LOD was determined as described in the figure caption for *Figure S2*. ECL micrographs for 125 µM [Ru(bpy)_3_]^2+^ (**Di**), 250 µM [Ru(bpy)_3_]^2+^ (**Dii**), 500 µM [Ru(bpy)_3_]^2+^ (**Diii**), and 750 µM [Ru(bpy)_3_]^2+^ (**Div**) solutions. In these experiments, the spheroids were first incubated in 250 µM TPrA and 4500 mg/L dextrose in a 1x DPBS solution for 15 minutes, then ECL was carried out in the varied concentration of [Ru(bpy)_3_]^2+^ and 4500 mg/L dextrose in 1x DPBS solutions.

**Figure S21.** **Amperometric it-curve corresponding to Figure 3.** Amperometric it-curve of the spheroid, which was first incubated in 250 µM TPrA and 4500 mg/L dextrose in a 1x DPBS solution for 15 minutes, then ECL was carried out in a 500 µM [Ru(bpy)_3_]^2+^ and 4500 mg/L dextrose in a 1x DPBS solution.

**Figure S22.** **Direct oxidation pathway for [Ru(bpy)_3_]^2+^ / TPrA ECL.** In this route, when the potential is applied, simultaneously, the [Ru(bpy)_3_]^2+^ and the TPrA are oxidized at the electrode surface to [Ru(bpy)_3_]^3+^ and TPrA^•+^, respectively. After the TPrA^•+^ is deprotonated to TPrA^•^, it then reduces [Ru(bpy)_3_]^3+^ to [Ru(bpy)_3_]^2+*^, which is in an excited state, while it itself is simultaneously oxidized to other products (P). The [Ru(bpy)_3_]^2+*^ then releases a photon with a *ca.* 620 nm wavelength as it relaxes back down to its ground state of [Ru(bpy)_3_]^2+^, being regenerated in the last step of this mechanism.

**Figure S23.** **Afterglow ECL intensity vs time plot and amperometric it-curve comparing a MCF-7 spheroid with the TPrA incubation versus a non-incubated MCF-7 spheroid.** **A.** Afterglow ECL intensity versus time plot, where t = 0 s represents the first frame of afterglow ECL (or the first frame after the cessation of the applied potential). The pink line represents a spheroid that was incubated in 250 µM TPrA and 4500 mg/L dextrose in a 1x DPBS solution for 15 minutes, while the blue line represents a spheroid that was not incubated. Both spheroids had ECL carried out in a 500 µM [Ru(bpy)_3_]^2+^ and 4500 mg/L dextrose in a 1x DPBS solution, and had 0.8 V vs Ag wire held for 180 seconds prior to the afterglow ECL, with the amperometric it-curves shown in **B**. The LOD was determined as described in the figure caption for *Figure S2*. The afterglow ECL was seen for about 90 seconds for the spheroid that was incubated in TPrA, while the non-incubated spheroid only had about 30 seconds of afterglow ECL. The camera parameters included an exposure of 500 ms and an EM Gain of 400.

**Figure S24. Amperometric i-t curves corresponding to the ECL micrographs in Figure 4a.** The spheroids interrogated were from the following cell lines: MRC-5, HEK-293, HPNE, Panc1, and MCF-7. Each spheroid was first incubated in 250 µM TPrA and 25 mM glucose in 1x DPBS solution for 15 minutes, then ECL was carried out in a 500 µM [Ru(bpy)_3_]^2+^ and 25 mM glucose in a 1x DPBS solution. 1.1 V vs Ag wire was held for 180 seconds.

**Table S2. Comparison of the ECL intensity from the last 15 frames of applied potential between spheroids from each type of non-cancerous to cancerous cell lines**.

| **Spheroid from a Non-cancerous Cell Line** | **Spheroid from a Cancerous Cell Line** | **Statistically Different ECL Intensities?** |
| --- | --- | --- |
| HEK-293 | Panc1 | YES  p value < 0.001 (***) |
| HEK-293 | MCF-7 | YES  p value < 0.05 (*) |
| HPNE | Panc1 | YES  p value < 0.0001 (****) |
| HPNE | MCF-7 | YES  p value < 0.001 (***) |
| MRC-5 | Panc1 | YES  p value < 0.01 (**) |
| MRC-5 | MCF-7 | YES  p value < 0.05 (*) |

**Figure S25. ECL Microscopy of a spheroid from the HPNE cell line without incubation in TPrA.** **A.** Brightfield micrograph of the spheroid and ECL micrographs of the spheroid at different time points during the experiment. The ECL was performed by placing the spheroid in an ITO cell containing 500 µM [Ru(bpy)_3_]^2+^ and 25 mM glucose within 1x DPBS, following the experimental set-up shown in *Figure S1*. **B.** ECL intensity vs time plot. The dashed gray line represents the limit of detection. To drive the ECL, a potential of 1.1 V vs Ag wire was applied for 180 s, as shown in the amperometric i-t curve in (**C**). After the cessation of the applied potential, only approximately 10 s of afterglow chemiluminescence was observed.

**Figure S26**. **Calibration curve of ECL intensity (a.u.) vs TPrA concentration (µM).** **A.** ECL intensity vs TPrA concentration calibration curve. The solutions consisted of 500 µM [Ru(bpy)_3_]^2+^, 25 mM glucose, and varied concentrations of TPrA (0 µM, 5 µM, 10 µM, 25 µM, 50 µM, 100 µM, and 250 µM) within 1x DPBS. 2 mL of each solution was loaded into an ITO cell (without a spheroid), following the same experimental set-up as shown in *Figure S1*. ECL was performed using an amperometric it-curve with 1.1 V vs Ag wire held for 3 minutes. For analysis, only the final 15 frames of ECL during the applied potential were averaged, with each concentration of TPrA having three separate trials. **B)** Corresponding, representative amperometric it-curves to each TPrA concentration interrogated in the calibration curve.

**Table S3. Estimated TPrA accumulation in spheroids derived from both cancerous and non-cancerous human cell lines based on their ECL intensities.**

| **Spheroid Human Cell Line**  **(^*^Indicates Cancerous Cell Line)** | **ECL Intensity (a.u.)**  **(Avg. (± St. Dev.))** | **Estimated TPrA Accumulation in Spheroid (µM)** |
| --- | --- | --- |
| HEK-293 | 206.3 (±4.1) | 52.7 (±1.0) |
| HPNE | 262.3 (±10.1) | 67.1 (±2.6) |
| MRC-5 | 219.6 (±34.1) | 56.1 (±8.7) |
| ^*^MCF-7 | 131.5 (±26.7) | 33.6 (±6.8) |
| ^*^Panc1 | 103.1 (±11.9) | 26.4 (±3.0) |

**SI Movie Descriptions**

**Movie S1 (separate file).** ECL microscopy of an ***MCF-7*** spheroid that was first incubated in 250 µM TPrA and 25 mM glucose in 1x DPBS solution for 15 minutes, then ECL was performed in a 500 µM [Ru(bpy)_3_]^2+^ and 25 mM glucose in 1x DPBS solution. Cyclic voltammetry was performed, with the CV swept from 0 to 1.3 V vs Ag wire at a scan rate of 50 mV/s. Corresponds to *Figure 1c*. The scale bar is 500 μm.

**Movie S2 (separate file).** ECL microscopy of an ***MCF-7*** spheroid that was first incubated in 250 µM TPrA and 25 mM glucose in 1x DPBS solution for 15 minutes, then ECL was performed in a 500 µM [Ru(bpy)_3_]^2+^ and 25 mM glucose in 1x DPBS solution. An amperometric i-t curve was performed, with an applied potential of 0.8 V vs Ag wire held for 180 seconds. Corresponds to *Figure 1b*. The scale bar is 500 μm.

**Movie S3 (separate file).** ECL microscopy of an ***MCF-7*** spheroid that was first incubated in 250 µM TPrA and 25 mM glucose in 1x DPBS solution for 15 minutes, then ECL was performed in a 500 µM [Ru(bpy)_3_]^2+^ and 25 mM glucose in 1x DPBS solution immediately after the incubation. Cyclic voltammetry was performed, with the CV swept from 0 to 1.3 V vs Ag wire at a scan rate of 50 mV/s. Corresponds to *Figure 2c(i)*. The scale bar is 500 μm.

**Movie S4 (separate file).** ECL microscopy of an ***MCF-7*** spheroid that was first incubated in 250 µM TPrA and 25 mM glucose in 1x DPBS solution for 15 minutes, then ECL was performed in a 500 µM [Ru(bpy)_3_]^2+^ and 25 mM glucose in 1x DPBS solution about 3.1 hours (or 11,200 seconds) after the incubation. Cyclic voltammetry was performed, with the CV swept from 0 to 1.3 V vs Ag wire at a scan rate of 50 mV/s. Corresponds to *Figure 2c(v)*. The scale bar is 500 μm.

**Movie S5 (separate file).** ECL microscopy of an ***MCF-7*** spheroid that was first incubated in 250 µM TPrA and 25 mM glucose in 1x DPBS solution for 15 minutes, then ECL was performed in a 500 µM [Ru(bpy)_3_]^2+^ and 25 mM glucose in 1x DPBS solution. An amperometric i-t curve was performed, with an applied potential of 0.8 V vs Ag wire held for 180 seconds. Corresponds to *Figure 3b*. The scale bar is 500 μm.

**Movie S6 (separate file).** ECL microscopy of an ***MRC-5*** spheroid that was first incubated in 250 µM TPrA and 25 mM glucose in 1x DPBS solution for 15 minutes, then ECL was performed in a 500 µM [Ru(bpy)_3_]^2+^ and 25 mM glucose in 1x DPBS solution. An amperometric i-t curve was performed, with an applied potential of 1.1 V vs Ag wire held for 180 seconds. Corresponds to *Figure 4a*. The scale bar is 500 μm.

**Movie S7 (separate file).** ECL microscopy of an ***HEK-293*** spheroid that was first incubated in 250 µM TPrA and 25 mM glucose in 1x DPBS solution for 15 minutes, then ECL was performed in a 500 µM [Ru(bpy)_3_]^2+^ and 25 mM glucose in 1x DPBS solution. An amperometric i-t curve was performed with an applied potential of 1.1 V vs Ag wire held for 180 seconds. Corresponds to *Figure 4a*. The scale bar is 500 μm.

**Movie S8 (separate file).** ECL microscopy of an ***HPNE*** spheroid that was first incubated in 250 µM TPrA and 25 mM glucose in 1x DPBS solution for 15 minutes, then ECL was performed in a 500 µM [Ru(bpy)_3_]^2+^ and 25 mM glucose in 1x DPBS solution. An amperometric i-t curve was performed with an applied potential of 1.1 V vs Ag wire held for 180 seconds. Corresponds to *Figure 4a*. The scale bar is 500 μm.

**Movie S9 (separate file).** ECL microscopy of a ***Panc1*** spheroid that was first incubated in 250 µM TPrA and 25 mM glucose in 1x DPBS solution for 15 minutes, then ECL was performed in a 500 µM [Ru(bpy)_3_]^2+^ and 25 mM glucose in 1x DPBS solution. An amperometric i-t curve was performed with an applied potential of 1.1 V vs Ag wire held for 180 seconds. Corresponds to *Figure 4a*. The scale bar is 500 μm.

**Movie S10 (separate file).** ECL microscopy of an ***MCF-7*** spheroid that was first incubated in 250 µM TPrA and 25 mM glucose in 1x DPBS solution for 15 minutes, then ECL was performed in a 500 µM [Ru(bpy)_3_]^2+^ and 25 mM glucose in 1x DPBS solution. An amperometric i-t curve was performed with an applied potential of 1.1 V vs Ag wire held for 180 seconds. Corresponds to *Figure 4a*. The scale bar is 500 μm.
